# The Mechanics of Building Functional Organs

**DOI:** 10.1101/cshperspect.a041520

**Published:** 2024-06-17

**Authors:** Toby G.R. Andrews, Rashmi Priya

**Affiliations:** The Francis Crick Institute, 1 Midland Road, London NW1 1AT, UK

## Abstract

Organ morphogenesis is multifaceted, multiscale and fundamentally a robust process. Despite the complex and dynamic nature of embryonic development, organs are built with reproducible size, shape, and function, allowing them to support organismal growth and life. This striking reproducibility of tissue form exists because morphogenesis is not entirely hardwired. Instead, it is an emergent product of mechanochemical information flow, operating across spatial and temporal scales – from local cellular deformations to organ-scale form and function, and back. In this review, we address the mechanical basis of organ morphogenesis, as understood by observations and experiments in living embryos. To this end, we discuss how mechanical information controls the emergence of a highly conserved set of structural motifs that shape organ architectures across the animal kingdom: folds and loops, tubes and lumens, buds, branches and networks. Moving forward, we advocate for a holistic conceptual framework for the study of organ morphogenesis, which rests on an interdisciplinary toolkit and brings the embryo centre stage.

## Introduction

How cells assemble into higher-order structures to build functional organs, critical for organismal life, has fascinated biologists and physicists for centuries (Baer 1828; Haeckel 1866; Thomson 1917; Vogt 1929; Peck 1953). During embryonic development, cells proliferate, differentiate, and rearrange to build simple tissue primordia, which evolve in form to adopt higher-order geometries like tubes, branches, loops, and ridges. These anatomical motifs sculpt complex organ architectures and confer them the function to sustain a growing organism. The mechanisms that drive morphogenesis *in vivo* operate across time and length scales and integrate diverse mechanochemical cues. Furthermore, the rich and ever evolving environment of a developing embryo generates unpredictable internal and external fluctuations. Yet, remarkably, growing embryos reproducibly build organs with the right shape, size, and function. In chick embryos, the heart loops with the correct orientation 97% of the time (Hoyle et al. 1992), and the vertebrate gut exhibits highly stereoscopical looping in a species-specific manner (Savin et al. 2011). This striking reproducibility of tissue shapes exists because morphogenesis does not have a hardwired blueprint, it is not a linear readout of genetic information. Instead, cells constantly adapt to surrounding cues to buffer against stochastic fluctuations and self-organise into a variety of macroscopic structures.

An organ is much more than the sum its constituent cells. The complex morphological patterns of organs emerge during development and cannot be deduced from its cellular and molecular constituents. Indeed, building functional organs requires dynamic inter-cellular and inter-tissue interactions and feedback from organ form and function. A developing heart will not form valves and trabeculae in the absence of blood flow and contractility (Gunawan et al. 2021). Foetal breathing movements are critical for lung development (Li et al. 2018). The last decade has also seen a significant realisation in the field that form cannot be explained exclusively by the dynamics of biochemical signalling networks. The notion that morphogenesis obeys physical rules, and needs to be understood beyond the molecular scale, is not new and was posited decades ago (Thomson 1917; Odell et al. 1981; Oster et al. 1983). The shape of an organ is inherently linked to the forces it experiences; from the behaviour of its constituent cells, neighbouring tissues, and the dynamic environment it is growing in (Nelson and Gleghorn 2012; Stooke-Vaughan and Campas 2018; Trepat and Sahai 2018; Hannezo and Heisenberg 2019; Hamant and Saunders 2020; Collinet and Lecuit 2021; Goodwin and Nelson 2021; Lenne et al. 2021; Maroudas-Sacks and Keren 2021). Thus, a synergy between biochemical signalling networks and mechanical forces is needed to explain the emergence of reproducible organ architectures *in vivo*.

Understanding how organ form and function emerge robustly is not only fundamental to the field of Developmental Biology, but is also essential to accelerate tissue engineering efforts and has significant implications for understanding birth defects (Good and Trepat 2018; Rossi et al. 2018; Dunwoodie and Wallingford 2020; Martyn and Gartner 2021; Veenvliet et al. 2021). To achieve this goal, it is imperative to understand how local cell- and tissue-scale deformations occur, and ultimately how these local deformations shape macroscopic organ architectures and influence their function. Because of the ease of experimental manipulations and optical accessibility, cell and tissue deformation dynamics have been studied extensively in *in vitro* systems and tissues with planar geometries (Bassel and Smith 2016; Fletcher 2016; Davies 2017; Saunders and Ingham 2019; Veenvliet et al. 2021). These systems are experimentally powerful and have significantly advanced our understanding of morphogenesis. Yet, our understanding of how complex 3D organ architecture and function emerge during embryonic development remains extremely limited. To be able to decode the robust nature of organogenesis, we need to study organs, as wholes, in living embryos, and move towards a more holistic approach that encompasses inter-tissue interactions, geometrical inputs, and feedback from function. Recent years have been transformative in terms of tools and techniques to visualize (Mickoleit et al. 2014; McDole et al. 2018; Wallingford 2019), manipulate and measure (Campas 2016; Sugimura et al. 2016; Gómez-González et al. 2020) morphogenesis. These technical advancements combined with increasing collaboration between biologists, engineers and theorists have made it possible to confront this grand challenge in developmental biology (Stooke-Vaughan and Campas 2018; Hannezo and Heisenberg 2019; Collinet and Lecuit 2021; Maroudas-Sacks and Keren 2021; Fletcher and Osborne 2022). And indeed, using modern experimental tools, there is a resurgence of studies striving to unravel the 4D (space + time) dynamics of organ morphogenesis as it unfolds inside the embryo (Mosaliganti et al. 2019; Priya et al. 2020; Tsai et al. 2020; Zhang et al. 2020; Fukui et al. 2021; Munjal et al. 2021; Palmer et al. 2021; Mitchell et al. 2022).

In this review, we will focus on the mechanical basis of organ morphogenesis, with a specific focus on visceral organs as they are the least understood (Morishita et al. 2017; Mitchell et al. 2022). We will discuss how intrinsic and extrinsic forces guide formation of a conserved repertoire of anatomical motifs that are critical for visceral organ function; from tissue folds and loops to lumen formation, and emergence of higher-order network topologies. Many parallels in organogenesis can be extracted from these diverse examples, as different organs across species use similar mechanisms to sculpt these anatomical motifs. And thus, rather than providing an exhaustive overview, we aim to highlight general design principles that build organs of diverse shape and size, across the animal kingdom. We conclude with open questions challenging multiscale understanding of organ morphogenesis *in vivo*. We argue that with a suite of powerful modern tools and theortical appoaches available to us, now is the best time to confront the complexity of embryonic morphogenesis and strive for a systems-level understanding of how form and function emerge during development.

## Building Folds and Loops

Tissue folds and loops shape various organs, from the fly wing disc (Tozluoǧlu et al. 2019) to vertebrate hearts (Desgrange et al. 2018). These motifs generate compartments and increase the tissue surface area to enhance material exchange during organogenesis. Interestingly, diverse organs across species seem to exploit conserved rules to build folds and loops, including differential growth induced mechanical instability, cell shape changes driven by apical or basal constriction, and mechanical interactions with surrounding tissues and the extra cellular matrix (ECM).

**Figure 1:**
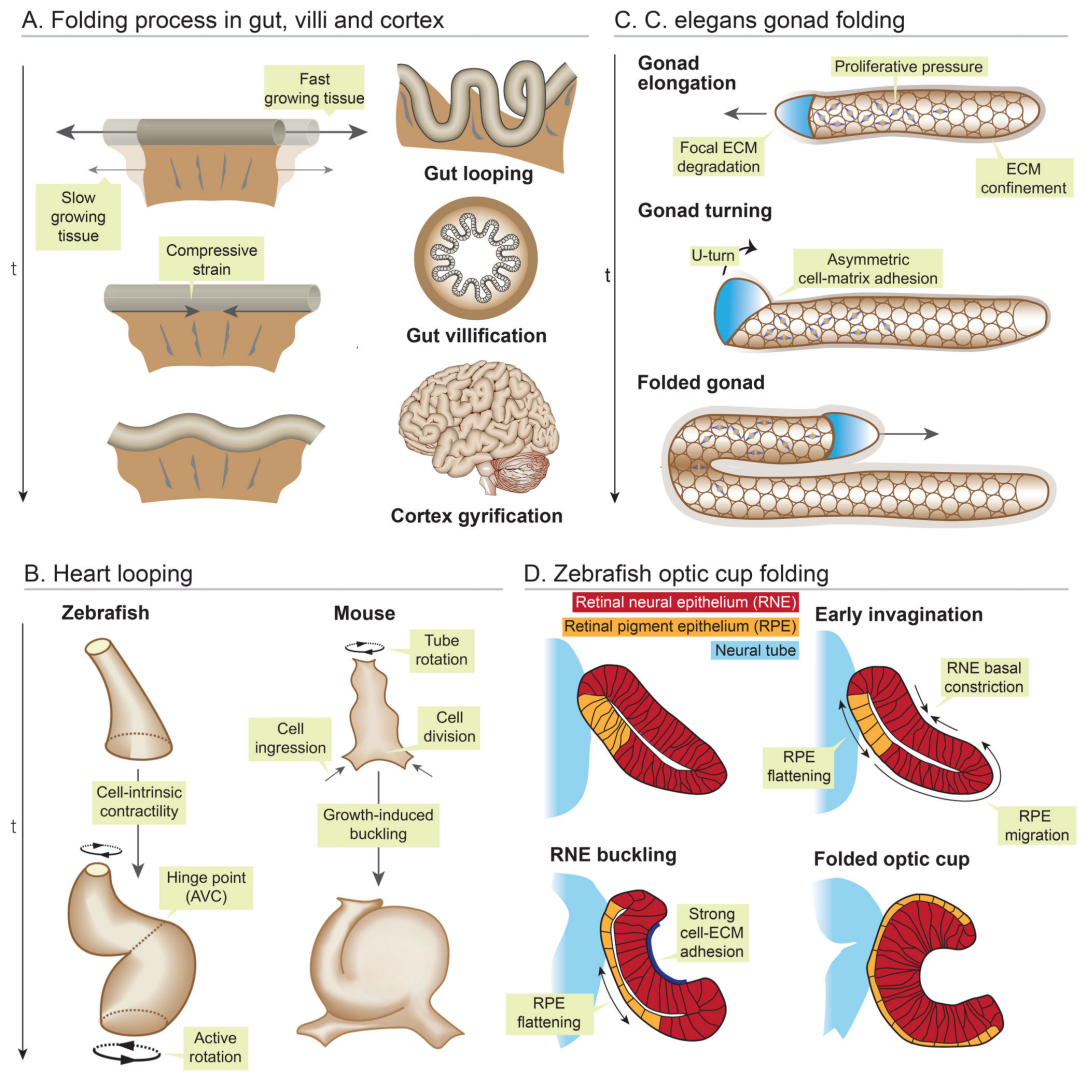
Physical principles of folding and looping morphogenesis. (*A*) A faster growing tissue confined by slower growing adjacent tissue experiences compressive forces, which generates mechanical instabilities, driving brain cortex folding, mid gut lopping and gut villification. (*B*) In zebrafish, actomyosin forces twists the two chambers of the heart around the atrioventricular (AV) canal, resulting in torsion of the linear heart tube into S-shape. In mice, asymmetric rotation, preferential cell ingression and proliferation at the ventral pole buckles the linear heart tube rightwards to form a loop. (*C*) In *C. elegans* proliferative pressure from germ cells and ECM confinement propels gonad elongation, while direction of folding is driven by polarised cell-ECM interaction. (*D*) Optic cup morphogenesis in zebrafish is a multifaceted process. RPE cells migrate and get incorporated into the inner RNE layer, which compresses and buckle the RNE. The basal surface of the RNE layer constricts and is constrained by ECM adhesion which further accentuates the buckling process. The outer RPE layer stretches and flattens to drive the optic cup folding.

Cortical folding is one of most striking architectural features of mammalian brains and has been associated with improved neural processing power (Striedter et al. 2015; Llinares-Benadero and Borrell 2019). These folds enable packing of a large cortical surface into a relatively small cranium (Tallinen et al. 2014). The process of cortical folding is complex and continues to elude biologists (Striedter et al. 2015; Llinares-Benadero and Borrell 2019). Several mechanical factors, including hydraulic pressure from secretion of cerebrospinal fluid and patterned axonal tension have been hypothesized to induce cortical folding (Striedter et al. 2015; Llinares-Benadero and Borrell 2019). An alternative hypothesis is that cortical folding emerges as a result of *differential tissue growth*, in which the outer grey matter grows faster than the inner white matter, thus compressing the cortex into folds (Richman et al. 1975) (Fig. 1A). MRI images of a 22-week-old smooth foetal human brain were used to create swelling-gel brain models with similar shape, size and material properties. When these gel brains were immersed in solvent, the outer layer swelled relative to the inner layer, recapitulating cortical growth and mechanical compression, ultimately yielding folds similar to human brain in wavelength and pattern (Tallinen et al. 2016). In addition to differential growth, tissue stiffness is an important factor governing this folding process. It has been shown that only when grey and white matter have similar stiffness levels, differential growth between them will yield folding patterns resembling the cortical folds of brain (Richman et al. 1975; Kaster et al. 2011; Tallinen et al. 2014), thus underlining the physical basis of brain folding.

A similar *growth induced mechanical instability* shapes the embryonic gut tube (Fig. 1A), which buckles into stereotypical number and size of loops to fit into the embryonic body cavity and maximize nutrient absorption. In chick, BMP induced differential growth drives this process. The rapidly elongating gut tube is confined by the slow-growing dorsal mesentery, thus undergoing mechanical compression induced folding (Savin et al. 2011; Nerurkar et al. 2017). Interestingly, the number and amplitude of gut loops in chick, quail, finch and mouse can be accurately predicted from the elastic properties of the gut tube, the elastic properties of the dorsal mesentery and differential growth parameters, thus emphasising the conserved role of mechanical instability in gut looping (Savin et al. 2011). Similarly, villi folding in the chick gut also relies on growth induced buckling (Fig. 1A) (Shyer et al. 2013). While in mice, BMP induced mesenchymal clusters deform and push the overlying epithelium into villi (Walton et al. 2016a, 2016b). For an in-depth reading on intestine and villi looping, please refer to these excellent reviews (Nelson 2016; Houtekamer et al. 2022)

Heart looping is another critical event during embryogenesis, driven largely by *buckling deformations* while integrating various *intrinsic and extrinsic cues* (Fig. 1B). The primitive heart is a relatively straight tube, which undergoes twists and turns to acquire an S-shape in fish or helix in chick and mouse (Taber 2006; Noël et al. 2013; Shi et al. 2014; Le Garrec et al. 2017; Desgrange et al. 2018). Molecular regulation of cardiac looping has been extensively studied and asymmetric gene expression patterns has been shown to regulate this process (Desgrange et al. 2018, 2020). However, the underlying biophysical mechanisms remain less understood. In Zebrafish, heart looping occurs as the linear tube twists around a fixed hinge, the atrioventricular canal (Fig.1B). The two chambers rotate in opposite directions thus twisting the tube into an S-shape (Tessadori et al. 2021). Strikingly, heart looping in zebrafish seems to be driven by cell-intrinsic actomyosin forces, given heart tubes cultured *ex vivo* retain their capacity to loop (Noël et al. 2013; Tessadori et al. 2021). While growth-induced buckling is not required for looping in this case, as ablating cell proliferation or recruitment does not prevent looping (Noël et al. 2013; Tessadori et al. 2021). In contrast, heart looping in mouse is driven by growth induced mechanical constraints and asymmetric tissue rotation (Le Garrec et al. 2017; Desgrange et al. 2018, 2020). The two ends of the linear heart tube rotate in opposite directions, while preferential cell ingression and proliferation of cells occurs at the ventral pole (Fig. 1B). These sequential asymmetries buckle the linear heart tube rightwards to form a loop (Le Garrec et al. 2017; Desgrange et al. 2018, 2020). In chick, it has been postulated that the elongating heart tube physically buckles into a C shape either by confinement pressure from the pericardial cavity (Taber 2006; Männer and Bayraktar 2014), or forces generated by asymmetric changes in cell shape and size (Shi et al. 2014). However, a recent study showed that heart looping in chick does not rely on proliferation or cell shape changes. Instead, actin dependent asymmetric cellular rearrangements generate left-right difference in tissue deformation, which bends the linear heart tube (Kawahira et al. 2020).

Tissue morphogenesis does not happen in isolation, and it is inevitable that the mechanics of the surrounding tissue and ECM interactions will influence folding deformations. Gonad folding in C. elegans is a striking example of folding event driven by *compressive pressure and asymmetric cell-ECM adhesion* (Fig. 1C) (Agarwal et al. 2022). Pushing forces generated by proliferating germ cells and ECM confinement pressurizes the distal tip cell (DTC). The pressurized DTC cell locally degrades ECM and propels itself forward to elongate the gonad. The growing DTC cell eventually take a U-turn to generate a fold, and this is driven by polarised cell-ECM interaction. The dorsal side of DTC exhibits enriched cell-ECM adhesion, which acts as a hinge point. As a result, the pressurized growing DTC experience a torque force and rotate along the adhesive axis, generating a folded gonad (Agarwal et al. 2022).

Optic cup morphogenesis in zebrafish is an excellent example of a buckling event guided by *inter-tissue interactions* (Norden 2023). The optic vesicle of zebrafish is a bilayered neuroepithelium consisting of an inner layer of retinal neural epithelium (RNE) and an outer layer of retinal pigmented epithelium (RPE) (Fig. 1D). This bilayered neuroepithelium buckles into a hemispheric shape, driven by *actomyosin-induced basal constriction, crowding induced compression, and flattening* of the outer RPE layer (Heermann et al. 2015; Nicolás-Pérez et al. 2016; Sidhaye and Norden 2017; Moreno-Mármol et al. 2021). The actomyosin machinery of the RNE cells undergo pulsatile contraction thereby progressively constricting their basal surface. These supracellular constriction forces are transmitted to the whole tissue via laminin-mediated attachment to the ECM, and disrupting this interaction affects folding events (Heermann et al. 2015; Nicolás-Pérez et al. 2016; Sidhaye and Norden 2017). Additionally, compressive forces generated within the RNE layer because of migration of cells from the presumptive RPE layer (called rim involution) bends the optic cup (Heermann et al. 2015; Sidhaye and Norden 2017). Recently, it was shown that the outer RPE cells reorganise their microtubule cytoskeleton to undergo flattening and cover the entire neural retina to contribute to this morphogenetic process (Moreno-Mármol et al. 2021). Thus, mechanical forces generated by RPE layer flattening complements the basal contraction and compressive forces of RNE to shape the optic cup, emphasizing the importance of inter-tissue interaction in tissue morphogenesis (Fig. 1D).

Similarly, heart valve formation is an intricate tissue folding event relying on *inter-tissue and ECM interactions*. In zebrafish, during atrioventricular valve formation, a subset of endocardium cells experiencing higher shear stress extend protrusions, undergo partial EndoMT (Endothelial-Mesenchymal Transition) and migrate into the ECM to form the primordial folded valve leaflet (Beis et al. 2005; Vermot et al. 2009; Gunawan et al. 2019; Chow et al. 2022; Gunawan et al. 2021; Vignes et al. 2022). The outflow tract (OFT) valve development relies on extensive crosstalk between endocardium and smooth muscle cells and mechanosensitive Piezo and Trp channels (Duchemin et al. 2019; Boezio et al. 2020). The endocardium invaginates into the ECM, followed by smooth muscle cells recruitment and ECM remodelling forming the folded structure of OFT valve (Duchemin et al. 2019; Boezio et al. 2020). Similarly, midgut folding in Drosophila is another example of how *inter-tissue mechanical interactions* can generate folds to shape internal organs (Mitchell et al. 2022). The midgut starts as a bilayer linear tube of an inner epithelial layer enveloped by smooth muscles. As the gut tube grows in length, it also constricts to acquire folded contours and form chambers. This folding process in instructed by the adjacent smooth muscle layer which undergoes calcium-induced patterned contraction, thus straining the underlying layer and inducing localised cell shape changes. These cell shape deformations induce organ-scale folding to shape the gut (Mitchell et al. 2022).

One emerging theme from these studies is that the exact pattern of folding, that is the number, position and spacing of folds, does not have to be specified by chemical cues and physical rules can explain the emergence of these patterns (Savin et al. 2011; Tallinen et al. 2016; Karzbrun et al. 2018). Yet, it is remarkable that these folding patterns appear robustly and reproducibly during embryogenesis. Tozluoǧlu et al. (2019) addressed this fundamental problem by computationally modelling Drosophila wing imaginal disc, which forms three folds at stereotypical positions. They found that while growth induced mechanical instabilities, tissue stiffness and basement membrane confinement are necessary for wing folding, it is differential growth that specifies the position of folds in the tissue. Considering that heterogeneity in growth and mechanics is one of the earliest and most described mechanism driving tissue buckling (Nelson 2016; Tozluoǧlu and Mao 2020), it is plausible to hypothesize that other systems might rely on this process to generate stereotypical folding patterns.

## Building Tubes and Lumens

Formation of tubes with fluid-filled lumens is essential for transmission of gases and fluids in diverse organ systems. During embryogenesis, tubular structures are built using a range of cellular strategies – de novo nucleation of a central lumen (Rasmussen et al. 2012; Akhtar and Streuli 2013; Buckley et al. 2013), cavitation through programmed cell death (Melnick and Jaskoll 2000); cell coalescence around pre-existing space (Medioni et al. 2008; Santiago-Martinez et al. 2008; Helker et al. 2013); folding, buckling or telescoping of epithelia (Nikolopoulou et al. 2017; Li et al. 2020).

**Figure 2:**
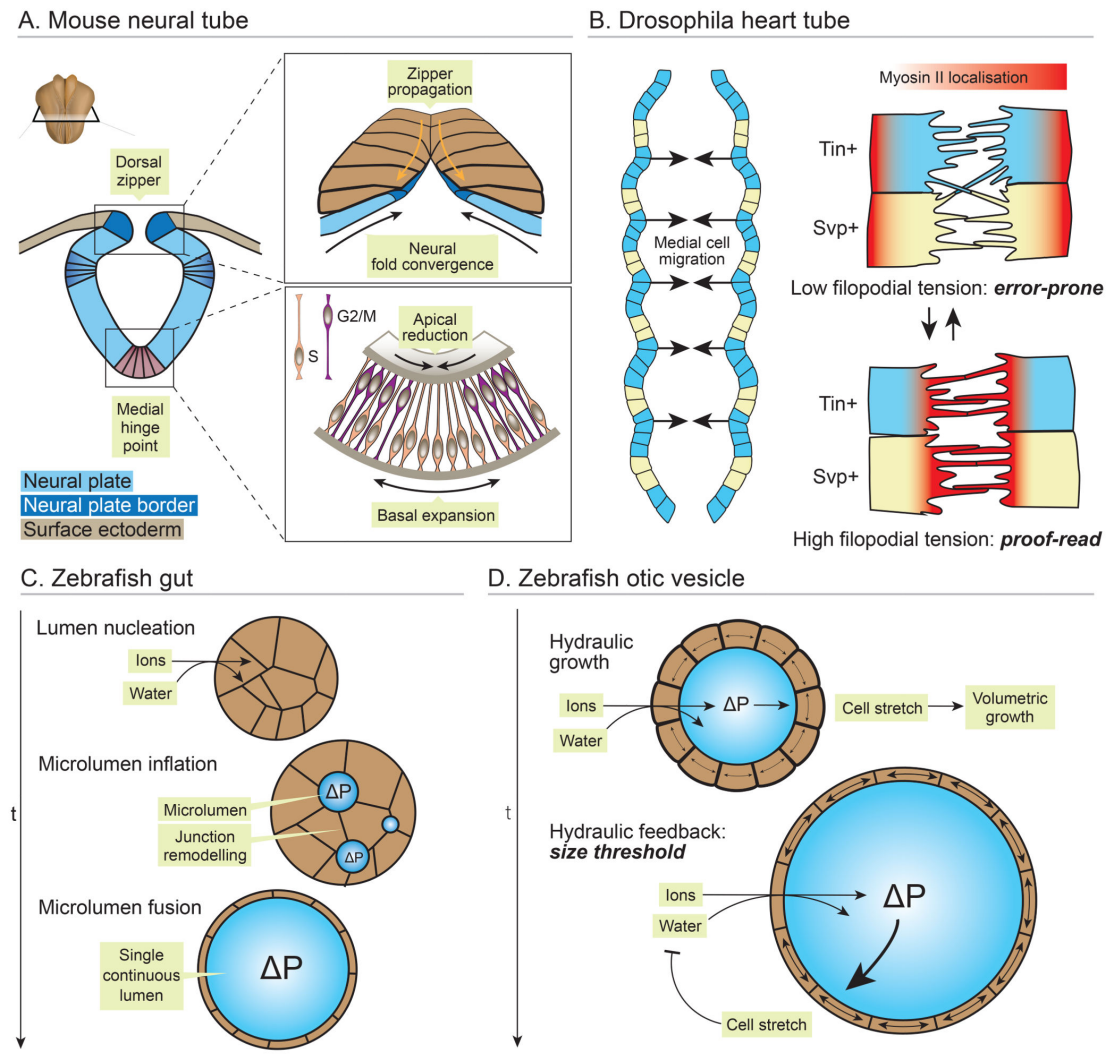
Physical principles of tube morphogenesis. (*A*) The vertebrate neural tube forms through folding of the epithelial neural plate. Schematic shows a transverse section of an amniote embryo, with the neural folds elevating and converging at the dorsal midline. (Top inlay) Cell junction contraction and remodelling zippers the neural folds together and separates them from the surface ectoderm. (Bottom inlay) Protraction of S-phase at the ventral midline prolongs basal nuclear localisation, driving medial hinge point formation. (*B*) The drosophila heart tube is formed by bilateral rows of cardioblasts (CBs) that converge at the dorsal midline and form new cell-cell adhesions. CBs extend filopodia that form homotypic contacts with contralateral CBs owing to differential adhesion profiles. Myosin II oscillates between the front and rear of each CB, operating a proof-reading system. At the rear, filopodia can extend and form nascent adhesions. At the front, filopodial tension increases, severing heterotypic adhesions and reinforcing homotypic adhesions. (*C*) The zebrafish gut forms a lumen de novo through cord hollowing. Active ion transport builds an osmotic gradient, followed by water. Positive hydraulic pressure (ΔP) inflates small microlumens, which fuse through junctional remodelling. (*D*) The zebrafish otic vesicle similarly inflates through generation of hydraulic pressure. As the lumen grows, surrounding cells stretch and undergo viscoelastic shape change. High hydraulic pressure inhibits ion transport, allowing self-organisation of lumen size.

Vertebrate neural tube closure is a classic example of tube formation via *epithelial folding* (Fig. 2A). Patterned cellular force production bends the initially flat neural plate, and formation of new intercellular junctions seals its free edges to define an epithelial tube. To achieve this, cells adopt a wedge shape, with reduced apical and expanded basal domains. Cell wedging is driven by apical actomyosin contractility in Xenopus (Haigo et al. 2003; Itoh et al. 2014), while in the amniote neural plate, cell shape is largely determined by nuclear position. In amniotes, signalling from the notochord delays cell cycle progression at the midline, which prolongs basal localisation of S-phase nuclei and thereby swells the basal pole of the cell (Smith and Schoenwolf 1988; Ybot-Gonzalez et al. 2002) (Fig. 2A). Apical actomyosin tension also resists apical expansion caused by apical migration of mitotic nuclei (Butler et al. 2019; Galea et al. 2021). As the neural folds elevate, a zippering mechanism, driven by waves of junctional contraction, rearrangement and relaxation, seals them together at the midline. In this process, the neural tube detaches from the non-neural ectoderm, and the more posterior neural folds are pulled medially for fusion (Hashimoto et al. 2015; Galea et al. 2017; Hashimoto and Munro 2019). Mechanical interfaces with surrounding tissues are also essential for neural tube closure; proper migration and convergent extension of paraxial mesoderm promote hinge point formation, while cell rearrangements and actomyosin dynamics in the surface ectoderm support posterior neural tube closure (Nikolopoulou et al. 2019; Zhou et al. 2020; Christodoulou and Skourides 2022; Li et al. 2022). Recently, micropatterned stem cells recapitulating human neural tube closure also hint at a role for basement membrane synthesis by the surface ectoderm for physical detachment of the neural plate and its subsequent folding (Karzbrun et al. 2021).

The Drosophila heart tube also forms through *cells reorganising* their positions and adhesive contacts to enclose existing space as a new lumen (Fig. 2B). In this case, two bilateral rows of cardioblasts (CBs) converge through the mass tissue movements of dorsal closure, and active medial migration across the ectoderm (Haack et al. 2014). Once in proximity of 15-20µm, CBs extend filopodial protrusions and establish contact with their specific contralateral partner, reliant on localised actin polymerisation (King et al. 2021). CBs first form new adhesions at their dorsal edge, then adopt a crescent shape to form adhesions at their ventral edge (Medioni et al. 2008). A lumen persists between these domains, owing to cell-ECM signalling and enrichment of repulsive Slit/Robo ligand-receptor signalling, which later provides a passage for transmission of haemolymph (Medioni et al. 2008; Santiago-Martinez et al. 2008). Crucially, new adhesions are formed with specificity between two distinct CB subtypes (Tin+ and Svp+) that organise in a repetitive pattern. Recent work identified differential adhesion profiles between CB subtypes that ensures robust pairing between homotypic cells (Zhang et al. 2018). The fidelity of cell matching is further improved by a mechanical proofreading system, owing to oscillatory localisation of Myosin II between the rear and leading edge of the cell (Fig. 2B). Myosin II enriches at the leading edge at 4-minute intervals, where it increases filopodial tension. This tension severs weak adhesion between heterotypic cells, while reinforcing adhesions between homotypic cells (Zhang et al. 2020). These dynamic cellular interactions and cell intrinsic tension therefore resolve molecular heterogeneities between cells into a robust tubular architecture.

The primitive gut tube is the primordium for the gastrointestinal tract and respiratory systems. In amniotes, the gut tube forms through *epithelial folding* (Tremblay 2010; Nowotschin et al. 2019). However, in anamniotes it forms through a different mechanism, termed *cord hollowing* (Bagnat et al. 2007). In cord hollowing, an initially dense cellular condensate follows symmetry breaking cues that polarize cells across a new inside-out axis, typically with cells facing their apical domains towards a new presumptive lumen (Sigurbjornsdottir et al. 2014). This lumen is then inflated through fluid influx, or deposition of extracellular matrix components as is the case for the Drosophila trachea (Tonning et al. 2005). In the zebrafish gut, activity of apical Na+/K+ ATPase builds a steep osmotic gradient across the epithelium, which is followed by paracellular influx of water (Fig. 2C). Crucially, the rate of fluid influx must be tightly controlled to ensure robust size control, where experimentally increasing the osmotic gradient leads to excessive lumen growth and epithelial rupture (Bagnat et al. 2007, 2010). Water influx into the gut first generates multiple small and spherical microlumens, separated by bridges composed of basolateral cell-cell contacts lacking apical and tight junction proteins, which prevent local fluid equilibration (Bagnat et al. 2007). Merging of microlumens requires active junctional remodelling, which shrinks basolateral contacts and delivers new apical proteins to the expanding lumenal surface (Alvers et al. 2014) (Fig. 2C). Thus, the force of fluid influx and junctional remodelling collectively assemble a single continuous lumen.

Another example of lumen formation through *hollowing* is the otic vesicle, which gives rise to a labyrinth of tubules in the inner ear responsible for auditory perception and balance. In zebrafish, the otic vesicle arises from a dense thickening of cells beneath the otic placode, which hollows and inflates with endolymphatic fluid (Haddon and Lewis 1996) (Fig. 2D). As fluid accumulates in the vesicle lumen, surrounding cells thin and reduce their volume, implying a net fluid movement from cells into the lumenal space (Hoijman et al. 2015). However, morphometric analysis in later developmental stages revealed extensive volumetric growth, primarily in the vesicle lumen, with only a minor increase in total cellular volume (Mosaliganti et al. 2019). This means additional influx of extracellular fluid is required for vesicle inflation. Like in the gut, fluid accumulates in the otic vesicle owing to an osmotic gradient established by cellular Na+/K+ ATPase activity. As demonstrated using a piezo-based tension sensor, this drives an increase in hydraulic pressure, which provides the force to stretch surrounding cells and inflate the lumen (Mosaliganti et al. 2019) (Fig. 2D). This depends on acute elastic cellular deformations, in addition to long term plastic shape change. However, high hydraulic pressure is ultimately inhibitory to fluid flux, leading to stabilisation of vesicle volume at a size threshold. As such, while ruptured vesicles first collapse, they eventually repair epithelial integrity and re-initiate fluid influx to restore their original volume (Mosaliganti et al. 2019). The otic vesicle thus demonstrates a self-organising mode of lumen size control governed by hydraulic feedback.

## Building Buds, Branches and Networks

### Cellular dynamics of budding and branching

To build organs, cellular primordia remodel and assemble into higher-order network architectures. Often such networks are tubular and convey fluids, while other cellular networks mediate efficient transfer of mechanical and electrical information (Gosak et al. 2022). The first challenge in understanding network growth is how new branches are positioned in organ primordia and remodelled to promote stable outgrowth. The second is what information steers growing networks to robust scale and topology, enabling optimal organ function. As explained in the following case studies, network growth demands localised control of forces within and between cells, and the ECM, and an information relay between local cellular dynamics and emerging organ function.

**Figure 3:**
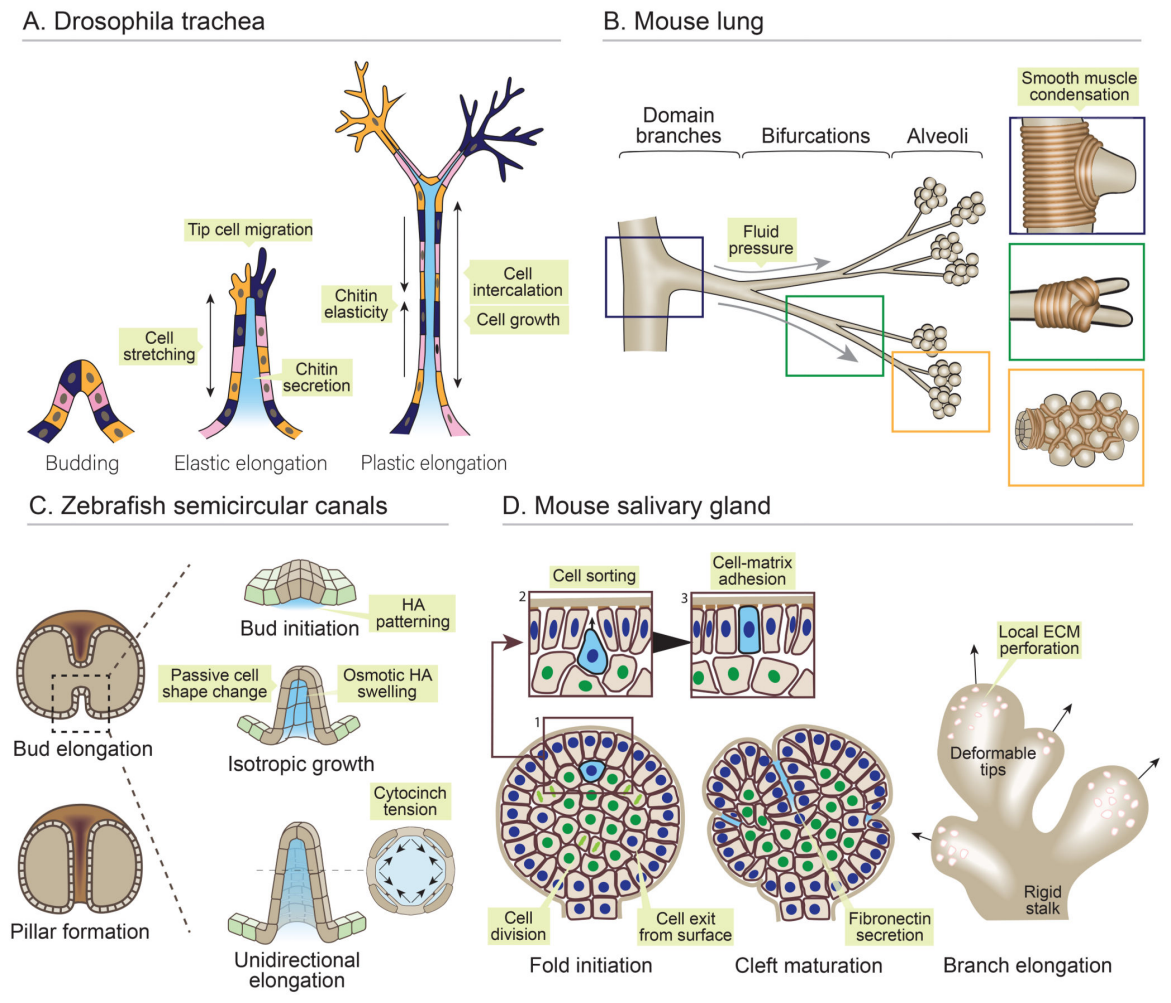
Morphogenesis of epithelial buds and branches. (*A*) The drosophila trachea branches through epithelial remodelling in post-mitotic cells. Buds elongate through active migration of tip cells, which increase tension in the stalk, leading to elastic cell stretch and branch outgrowth. Area growth and intercalation of stalk cells enables plastic elongation, restricted by elasticity of a lumenal chitin matrix. (*B*) Domain branches, bifurcations and alveoli in the mouse lung are sculpted by smooth muscle fibres which locally condense at branch tips and form a physical growth barrier. The airway epithelium buds between smooth muscle fibres under positive hydraulic force. (*C*) Zebrafish semi-circular canals appear as buds extending into the otic vesicle lumen. Hyaluronic acid (HA) is secreted locally beneath the prospective bud, and osmotically swells, leading to budding through passive cellular deformations. Cytocinches between cells increase circumferential tension, translating isotropic HA swelling into anisotropic bud elongation. (*D*) The mouse salivary gland buds through folding of the epithelial surface and basement membrane. Cells exit the surface layer (blue nuclei) and divide in the core of the bud (green nuclei). Daughter cells (blue cell) then sort from inner cells owing to differential adhesion, return to the outer, and restore adhesion with the basement membrane. Growth of the surface layer folds the basement membrane, forming clefts stabilised by fibronectin secretion. Outgrowth of new branches is aided by focal ECM degradation, converting it from a stiff shell to a deformable lattice.

The Drosophila trachea is a highly ramified network for cellular gas exchange, whose formation depends on local force transmission between cells. A striking feature of this network is its morphogenesis in the absence of cell division, indicating a dominant role for *cell reorganisation* (Samakovlis et al. 1996). Tracheal cells first become distinct as sac-like evaginations of surface ectoderm, which branch in a deterministic pattern to invade surrounding tissue. Primary branches of the tracheal system arise through active chemotactic migration of 1-2 tip cells (Fig. 3A). As they migrate, tip cells generate traction force and pull a stream of stalk cells, tightly connected by adherens junctions, from the tracheal sac. Branch elongation requires elastic deformations in stalk cells, shown by their rapid retraction to a resting state upon tip cell laser ablation (Caussinus et al. 2008). The stalk also exhibits long-term plastic deformation owing to myosin II-independent radial cell intercalation, induced by the tension arising from tip cell migration (Ochoa-Espinosa et al. 2017) (Fig. 3A). In the trachea, the morphology of maturing branches is constrained by a lumenal chitin matrix (Tonning et al. 2005). Elasticity of chitin antagonises tube elongation driven by growth of apical cell areas (Dong et al. 2014). In response to lumen growth through chitin secretion, cells also assemble actomyosin in a circumferential orientation, whose contractility restricts excessive lumen growth (Hannezo et al. 2015; Ozturk-Colak et al. 2016). Thus, branch geometry arises as a dynamic balance of forces between cells and their secreted ECM.

Branching of the mouse lung does not require intrinsic cellular force production, and instead depends on *patterned physical constraints*. The lung begins as a hollow wishbone-shaped primordium, which invades a mass of pulmonary mesenchyme through rhythmic cycles of elongation and branching (Goodwin and Nelson 2020). As the epithelium grows, it is enveloped by a stiff cage of smooth muscle, which acts as a physical growth barrier (Fig. 3B). New domain branches form by protruding through gaps in the cage, positioning the major lobes of the lung. Further condensation of smooth muscle defines clefts that subdivide branches and propagate a space-filling tree (Metzger et al. 2008; Kim et al. 2015; Goodwin et al. 2019). Similarly, corrugations in the lizard lung arise from the airway epithelium being pushed through a rigid hexagonal mesh of smooth muscle (Palmer et al. 2021). Recently, it was shown that alveoli – the gas exchange units of the lung – also arise through pouching of cells through a ring of smooth muscle cells (Gillich et al. 2021). This occurs during a critical fate decision between alveolar type I (AT1) and type II (AT2) progenitors. Mesenchymal FGF signalling selects a single AT2 progenitor, which sensitises its neighbours to stretch and flatten under the hydrostatic pressure generated by foetal breathing movements (Gillich et al. 2021; Brownfield et al. 2022). This physical deformation promotes AT1 identity (Li et al. 2018; Shiraishi et al. 2023), and forces cells to bud through the smooth muscle ring and generate a dome-shaped alveolus (Gillich et al. 2021) (Fig. 3B). At each scale, branch position is thus dictated by local mechanical heterogeneities arising from inter-tissue interaction. Once formed, mechanical forces play crucial roles in branch elongation. Longitudinal tension biases cell division orientation (Tang et al. 2018), and shear forces from fluid flux have been implicated in anisotropic cell shape changes that support directional growth (Conrad et al. 2021).

The zebrafish semicircular canal system is a remarkable case of epithelial budding through *extracellular force production*. The inner ear of jawed vertebrates contains three semicircular canals (SSCs), where fluid displacement excites sensory hair cells to detect body motion and orientation. In zebrafish, the SSCs are assembled from invaginations in the wall of the otic vesicle that project into its central cavity and fuse to form three tubular pillars (Fig. 3C). This budding process occurs in the absence of active cellular rearrangements or localised cell division (Munjal et al. 2021). Instead, a dense ECM rich in the polyelectrolyte hyaluronan assembles beneath the bud and expands in volume through water absorption (Munjal et al. 2021) (Fig. 3C). This osmotic swelling stretches the overlying cells into a dome shape, thereby initiating bud formation. Although cells do not exert active shape change through actomyosin contractility, E-cadherin rich intercellular tethers termed cytocinches generate anisotropic tension across the circumference of the bud. This circumferential tension resists radial bud dilation, and therefore translates isotropic growth driven by hyaluronate swelling into anisotropic tube elongation (Munjal et al. 2021). Thus, cellular processes align extracellular forces to enforce directional tissue-scale shape change.

The mouse salivary gland generates a branched pattern through *dynamic cellular interactions with the ECM*. The salivary gland begins as a stratified epithelial bud wrapped in a stiff layer of basement membrane (Fig. 3D). For the bud to build a branched network, clefts arise within its surface that partition cells into distinct territories that will form branches (Harunaga et al. 2011). Clefting initiates with addition of new cells to the surface epithelial layer, which promotes inward folding owing to tight adhesion with the juxtaposed basement membrane. Rather than translocating from the epithelial core of the bud, these new cells originate in the surface layer, then transiently move to a subsurface position, where they divide before reinserting into the surface layer (Wang et al. 2021). Robust reinsertion of daughter cells relies on differential E-cadherin adhesion, which enables local sorting of daughter cells from neighbouring interior cells, and their rapid movement between surface cells to restore anchorage to the basement membrane. As the epithelium folds, clefts that separate new branches penetrate the bud owing to focal deposition of fibronectin (Fig. 3D). While budding requires cellular adhesion to a stiff basement membrane, subsequent branch elongation demands its active softening. This is achieved by formation of bleb-like protrusions and focal secretion of protease enzymes, which remodel the basement membrane from a stiff shell to a deformable lattice that permits elongation while maintaining cell cohesion (Harunaga et al. 2014).

### Strategies for network topology control

**Figure 4:**
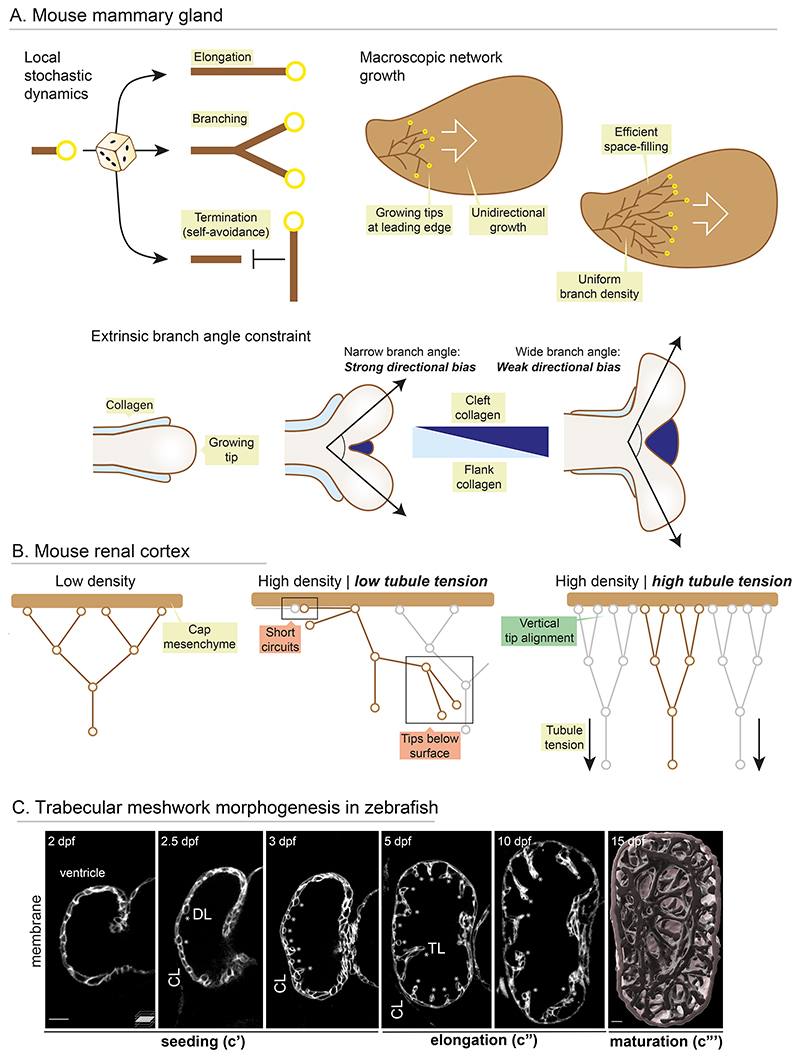
Mechanical control of network growth dynamics. (*A*) The mouse mammary gland epithelium branches with stochastic dynamics. (*Left*) Branches make stochastic decisions to elongate or branch and terminate through local self-avoidance. (*Right*) These dynamics allow robust network growth with uniform density, effectively filling space. (*Bottom*) Collagen accumulates on the flanks of branches and limits branch angle. Branch angle is defined by relative collagen enrichment in the flank and cleft, and dictates global network directionality. (*B*) In the mouse renal cortex, branch tips interface with the cap mesenchyme for nephron induction. At high density, this topology can be disrupted, with tips short-circuiting or falling beneath the surface. Tubule tension ensures a vertical alignment of tip families at high density, allowing a high packing density with robust mesenchymal interfaces. (*C*) Trabecular meshwork morphogenesis in zebrafish. Mid-sagittal section (c’, c”) and 3D surface rendered (c”’) images of zebrafish hearts expressing membrane marker. Compact layer (CL), delaminating, (DL, asterisks) and trabecular (TL, asterisks) cells. Stochastic single cell delamination from the outer compact layer seeds trabecular layer cells, and this seeding is triggered by mechanical heterogeneity (c’). These single trabecular cells transform into multicellular ridges (c”), and these ridges remodel and coalesce to form a mature 3D topological meshwork filling the ventricle lumen (c”’). Scale bar = 50 μm.

On a macroscopic scale, network topologies of visceral organs must be under robust developmental control. While branching patterns in the early mouse lung and kidney are highly stereotyped (Xu et al. 2017; Menshykau et al. 2019), the ductal network of the mouse mammary gland lacks stereotypic branching dynamics, where the probability of bud branching or termination is equal throughout the network (Scheele et al. 2017). This has led to a proposal that higher-order network topology emerges from *local stochastic rules* governing whether a bud will extend or branch, and *local self-avoidance* to terminate branch growth at high density (Hannezo et al. 2017) (Fig. 4A). Network growth generated by these principles enables topological self-organisation and space-filling, thus generating wide variation in organ geometry. Variations in starting conditions, the sensitivity of self-avoidance, and the relative contribution of global guidance cues, can also give rise to organ-specific network growth dynamics and topological motifs (Hannezo et al. 2017; Palavalli et al. 2021; Ucar et al. 2021). However, recent work suggests mechanical information can also sway network growth dynamics to bias higher-order properties. In mammary branching, collagen filaments assemble along the flank of elongating buds, which elevates local stiffness and limits branch bifurcation angle, thereby imposing a net directional bias in network alignment despite lack of global guidance (Nerger et al. 2021; Ucar et al. 2021) (Fig. 4A). A recent study in the kidney similarly found mechanical cues to bias inherently noisy network growth dynamics. At high density, terminal branch tips enter an unstable state, from which tips can collapse below the surface or collide and short-circuit (Fig. 4B). However, tension on renal tubules biases tips to an optimal vertical alignment that enables stable mesenchymal interfaces for nephron induction (Prahl et al. 2023).

Once formed, tubular networks further remodel to enhance physiological efficiency, and fluid forces play an essential role in this remodelling. For example, in the developing retinal vasculature, network efficiency is improved by pruning redundant branches in response to blood flow. The sheer force of blood polarises endothelial cells against the flow of blood, resulting in retrograde migration from poorly perfused vessels to those supporting greater flow (Franco et al. 2015). Mathematical modelling has also predicted that antagonism between VEGF signalling and shear forces mediates a switch from sprouting angiogenesis to vascular remodelling in response to blood flow, thereby adapting network topology to physiological load (Barbacena et al. 2022). A similar remodelling based on fluid flux has been proposed for the pancreas. In the pancreas, the early branching network is noisy and inefficient, with extensive redundant links and inhomogeneity in tubule diameters. However, this resolves towards an explicit hierarchy of duct diameters converging towards a central drainage duct, owing to specific removal of redundant ducts. Importantly, while pancreatic cells can form topological networks in 3D culture, these networks fail to prune redundant links. This difference has been attributed to absence of fluid flux *in vitro*, which may therefore be an instructive cue for network optimisation *in vivo* (Dahl-Jensen et al. 2018). Thus, network architecture arises from multi-scale information flow, integrating local cellular dynamics and emergent organ-scale physiology.

Unlike the tubular networks discussed above, the trabecular meshwork of heart is composed of multicellular muscular ridges spanning the ventricle lumen (Fig. 4C). Trabeculae increase the muscle mass of the embryonic heart, ensuring efficient blood flow, electrical conduction and nutrient exchange (Samsa et al. 2013; Gunawan et al. 2021). Proper assembly and maturation of trabecular ridges is crucial, given embryonic lethality in vertebrate models where their density or topology are disrupted (Samsa et al. 2013; Gunawan et al. 2021). While the genetic pathways underlying trabeculation have been well-studied, how these structures are shaped in a developing heart remain elusive. The excellent tractability of zebrafish has proven to be instrumental in filling this knowledge gap. In zebrafish, trabecular cells emerge through delamination from the initially monolayered myocardium (Liu et al. 2010; Staudt et al. 2014; Priya et al. 2020). Using live imaging, mosaic genetic tools and biophysical measurements, it has been shown that seeding is triggered by local mechanical heterogeneities; proliferation within the myocardial layer builds a compressive strain and generates local differences in actomyosin-driven tension (Priya et al. 2020). A subset of cells of higher tension apically constrict and delaminate towards the lumen to seed the trabecular layer in a stochastic pattern (Fig. 4c’) (Priya et al. 2020). These single cells then build multicellular trabecular ridges, and these ridges remodel and coalesce to form a complex 3D topological meshwork, filling the lumen of ventricle and thickening the myocardial wall (Fig. 4c”, c”’). While seeding is inherently stochastic, the mature network appears to have stereotypic features in terms of ridge spacing and density (Fig. 4c”’). Importantly, blood flow, heart function, interaction with the adjacent endocardium layer and ECM dynamics are important for trabecular morphogenesis in both mouse and zebrafish (Liu et al. 2010; Peshkovsky et al. 2011; Samsa et al. 2013; Staudt et al. 2014; Jimenez-Amilburu et al. 2016; Rasouli and Stainier 2017; Del Monte-Nieto et al. 2018; Priya et al. 2020; Gunawan et al. 2021; Qi et al. 2022; Grego-Bessa et al. 2023). Moreover, multi-colour clonal analysis with Brainbow revealed a high degree of clonal heterogeneity in individual ridges (Gupta and Poss 2012), suggesting an important role for cell rearrangement in steering network maturation. This begs the question of what cellular dynamics shape ridges, and what mechanochemical cues guide the network to stereotypical topological features that enable optimal function. Of note, form follows function during heart development (Gunawan et al. 2021) and thus there is a great need to study these dynamic processes in a living embryo.

## Outlook

**Fig. 5:**
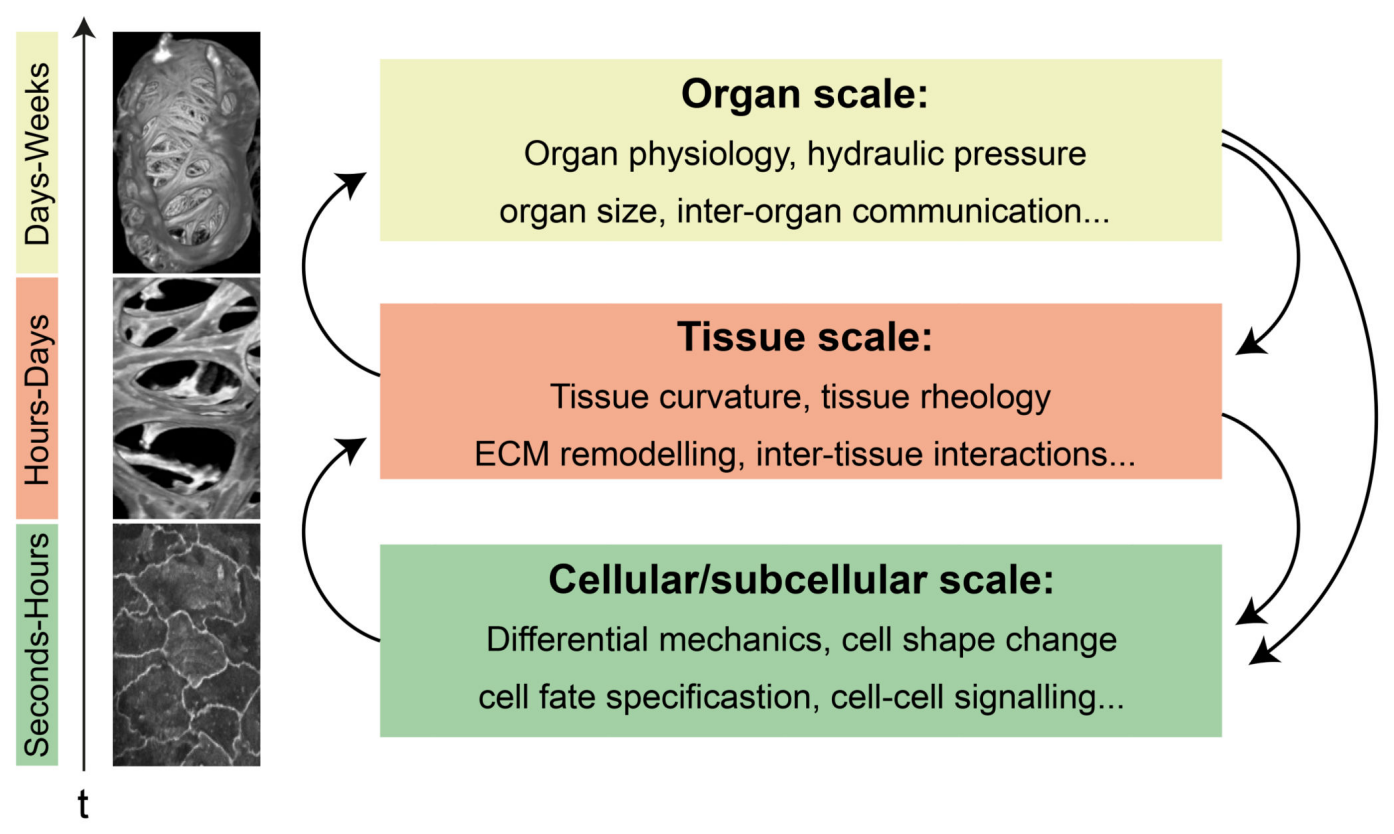
Bridging scales in Organ Morphogenesis. The robust shape and function of organs emerge through reciprocal mechanical interactions operating across spatiotemporal scales: from seconds to weeks, and between cells to tissues to organs and back.

Embryonic morphogenesis is an engineering marvel, while our capacity to engineer physiologically relevant tissue architectures *in vitro* is still limited. For example, various organoid models can recapitulate cell fate, but are still limited in their ability to reproduce the correct tissue forms and functions observed *in vivo* (Rossi et al. 2018; Hofer and Lutolf 2021; Veenvliet et al. 2021). Indeed, reconstituting complex motifs discussed in this review, which emerge robustly and reproducibly in embryos is still a major engineering challenge *in vitro* (Rossi et al. 2018; Hofer and Lutolf 2021; Martyn and Gartner 2021; Veenvliet et al. 2021).To fill this knowledge gap, we need to study morphogenesis in embryos in real time and ask what mechanisms cells deploy to sculpt tissues reproducibly and robustly. Here, a programme of rigorous observations, measurements and experiments will provide insights into *self-organizing mechanisms, emergent behaviour, and boundary conditions* which are fundamental to the robust nature of embryonic morphogenesis. Further, we need to move beyond the gene-centric understanding of development and conceptualize morphogenesis as an emergent product of mechanochemical information flows across spatial and temporal scales (Fig. 5). With this rationale, probing complex morphogenesis in embryos presents a host of technical and conceptual challenges. In this section we will discuss what we think are the next frontiers and open questions pertaining to the study of complex morphogenesis *in vivo*.

### Studying mechanics in vivo

A major challenge in studying biomechanics during tissue morphogenesis *in vivo* is the measurement and manipulation of forces across organizational scales. This expands to the material, or rheological properties of the growing tissues, which have emerged as key regulators of tissue shape, size and growth dynamics (Davidson 2011; Petridou and Heisenberg 2019; Maroudas-Sacks and Keren 2021). Yet, measuring and manipulating mechanical and material properties *in vivo* is not trivial (for a comprehensive read, see excellent reviews (Campas 2016; Sugimura et al. 2016; Petridou and Heisenberg 2019; Gómez-González et al. 2020). Because of their accessibility and simpler structure, embryonic surface tissues, tissues with planar geometries or *in vitro* systems are amenable to mechanical measurements and manipulation using various techniques. However, adapting these tools to perturb and measure forces and tissue rheology in a developing 3D tissue with spatial precision, and without disrupting the native physiological environment is an enduring challenge. Nevertheless, the renewed interest in *in vivo* mechanics has led to advancement of various techniques holding promise for application in complex 3D systems; for example, deformable droplet probes, force inference methods based on cell shape and geometry, genetically encoded FRET tension sensors, flipper membrane tension probes and Brillouin microscopy (Campas 2016; Sugimura et al. 2016; Petridou and Heisenberg 2019; Prevedel et al. 2019; Gómez-González et al. 2020). Although these techniques are still far from becoming routine workhorse tools for developmental mechanics labs, they are being constantly adapted and standardized for usage in a variety of model systems (Maitre et al. 2016; D'Angelo et al. 2019; Träber et al. 2019; Priya et al. 2020; Wang et al. 2020; Fukui et al. 2021; Moreno-Mármol et al. 2021; Shu et al. 2022; Stower et al. 2023). Ultimately, these tools will need to integrate with imaging and measurement of cellular behaviours and tissue-scale deformation dynamics *in vivo*; another challenge for deep, dense and 3D visceral organs. However, rapid developments in microscopy, deep learning and AI based image analysis will be instrumental in tackling this challenge (see a recent issue in Nature Methods 2023).

### Form, Forces and Fate

A crucial early step during organ development is establishment of diverse cell identities, which organise into complex spatial patterns. While extensive studies in the past have focussed on molecular control of cell fate (Gilmour et al. 2017; Briggs et al. 2018; Wagner et al. 2018; Pijuan-Sala et al. 2019; Kicheva and Briscoe 2023), in many developmental systems, cells differentiate while they or the tissues they inhabit are changing its shape and size (Maitre et al. 2016; Barone et al. 2017; Shaya et al. 2017; Lancino et al. 2018; Miroshnikova et al. 2018; Tang et al. 2018; Xia et al. 2019; Priya et al. 2020; Hadjivasiliou and Hunter 2022; Dullweber and Erzberger 2023). Moreover, there is now direct evidence that morphogenetic transitions can precede fate acquisition. For example, during trabecular morphogenesis in the zebrafish heart, cell fate specification follows morphogenesis. Mechanics-induced cardiomyocyte delamination is necessary and sufficient to trigger differential apicobasal polarity and Notch activation in the adjacent cells, thus generating two distinct tissue layers (Priya et al. 2020). Similarly, alveolar epithelial cell fate specification is preceded by changes in cell shape and mechanics (Tang et al. 2018). Thus, we need to devise experimental approaches to delineate the effect of mechanics on cell fate in a quantitative manner. One approach will be to derive a precise temporal correlation between cell fate, tissue mechanics and morphogenesis by performing live imaging of fast and dynamic cell fate reporters (Regot et al. 2014; Bothma et al. 2018; Wilcockson et al. 2023). Further, ectopic controlled manipulation of cell mechanics and signalling using optogenetics (Barone et al. 2017) or genetic mosaics (Maitre et al. 2016; Priya et al. 2020) will yield insight into the hierarchy of these induction events. Theoretical models incorporating cell shape, mechanics, and signalling status will also provide a holistic understanding of how these processes cooperate to generate diverse cell fates during morphogenesis (Chan et al. 2017; Shaya et al. 2017; Fletcher and Osborne 2022; Dullweber and Erzberger 2023).

### The issue of tissue geometry

During development, naïve biological tissues adopt intricate 3D geometries like curvature which is a ubiquitous feature of embryonic development. Thus, while most of our knowledge of morphogenesis comes from planar *in vitro* and *in vivo* systems, organs are inherently curved. Traditionally, formation of tissue curvatures has been attributed to localized cellular behaviours, like proliferation gradients and cell shape changes (Metzger and Krasnow 1999; Auman et al. 2007; Varner and Nelson 2014). Yet, in certain developmental contexts, formation of curvature precedes these localized cellular behaviours prompting us to re-evaluate this notion (Nogawa et al. 1998; Ingber 2005; Varner et al. 2015). For example, during lung branching morphogenesis, proliferation is locally patterned only after branch tips have acquired their characteristic curved shape (Varner et al. 2015). Positive tissue curvature defines the site of highest ERK activity in lung epithelium (Hirashima and Matsuda 2024). Of note, there is an increasing realization that curvature can dictate how mechanical stress is patterned, how signalling gradients are established and how local cellular behaviours are modulated (Nelson et al. 2006; Saw et al. 2017; Chen et al. 2019; Messal et al. 2019; Silver et al. 2020; Collinet and Lecuit 2021; Luciano et al. 2021). Yet, how geometry constrains cellular process to orchestrate morphogenesis remains poorly understood. However, innovative experimental approaches are rendering curvature a tractable property *in vivo*. In a recent study examining Drosophila gastrulation, the authors used a Fat2 mutant to alter the curvature of embryo, giving novel insight into how geometry instructs polarised tissue flows (Gehrels et al. 2023). In another study on Drosophila neck morphogenesis, the authors used inventive imaging methods to induce surface flattening to show that curvature acts in concert with homeotic gene expression to drive folding dynamics (Villedieu et al. 2023). Still, dissecting the role of curvature *in vivo* remains a complex challenge, where devising a tool that only perturbs curvature without any other pleiotropic effects is improbable. In turn, the accessibility and tractability of synthetic organoid and embryoid systems have proven to be extremely powerful in revealing the role of geometric complexity in morphogenesis (Ishihara et al. 2022; Vercurysse et al. 2022; Huang et al. 2023). Moving forward, an important goal should be to carefully analyse and interpret cellular processes in time and space in the context of tissue geometry.

### Scale Matters

Sculpting robust organ architecture and function requires integration of mechanisms operating at protein scale to the whole organismal scale, and from milliseconds to days (Fig. 5). At what organisational scale is complex morphogenesis best described, studied, and modelled? This question is not trivial to answer, especially given mechanical and biochemical signals operate and feed back across organizational length scales (Davidson et al. 2009). For example, activity and organization of cytoskeletal filaments determines cellular contractility, cell shape and rheology. Tissue scale mechanics originates from actomyosin supracellular dynamics, ECM, and inter-tissue interactions. And at the organ scale, geometrical constraints and physiological function influence global mechanical properties, which can in turn affect local cellular behaviour. While such non-hierarchal morphogenetic systems are robust, owing to layers of inherent feedback regulation, they also make inference of process and causality a grand challenge to developmental biologists and theorists (Davidson et al. 2009; Maroudas-Sacks and Keren 2021; Fletcher and Osborne 2022). Indeed, many efforts towards understanding organ morphogenesis have focussed on one scale of observation, for example gene expression or single-cell behaviours like shape changes, proliferation and death. While informative, these local properties need to be contextualised in the global context of organ form and function to understand the complex self-organised dynamics underlying higher-order tissue patterning. This feat is still not trivial and will require development of both novel experimental tools and strong interdisciplinary collaborations.

Theoretical modelling has proven enormously powerful in predicting and simulating developmental dynamics in complex systems, particularly for genetic patterning networks operating in the absence of cell rearrangement or tissue growth, like Drosophila germband segmentation (Jaeger et al. 2004; Clark 2017; Verd et al. 2018). In these cases, models question the sufficiency of known components to explain observed phenomena, predict emergent and non-intuitive dynamics, and clarify core regulatory features against redundancy and noise. Increasingly, modelling approaches are emerging to tackle the multi-scale nature of development. This includes vertex models to ask how form arises from local cellular force production and the physics of cell-cell interfaces (Alt et al. 2017), which have evolved to consider 3D epithelial geometries (Latorre et al. 2018; Okuda et al. 2018) and paved the way for modelling of epithelial shells and cell aggregates using active surfaces (Torres-Sanchez et al. 2022; Khoromskaia and Salbreux 2023). Now, a systems view of tissue morphodynamics is emerging, with the modelling of inter-tissue mechanochemical interactions, and mapping of gene regulatory interactions dynamics into physical modelling environments and live imaging data (Tozluoglu et al. 2019; Nematbakhsh et al. 2020; Uriu et al. 2021; Fulton et al. 2022). Looking forward, we believe that a holistic understanding of morphogenetic systems requires further development of experimental and theoretical methods that integrate gene expression and signaling with mechanical forces and physical deformations of cells and tissues. More importantly, however, it requires a meaningful synergy of theory and experiment throughout project development.

### The search for general principles

As developmental biologists continue to reveal details of tissue morphogenesis, one important question to ask is whether we can derive general principles for the sculpting of tissues and organs. In some terms, general mechanisms exist in biology. For example, Notch signalling is used repeatedly in various development contexts to drive cell fate decisions through lateral inhibition (Bray 2016; Dullweber and Erzberger 2023). The expression of *tinman* and its vertebrate homologue *Nkx2.5* is conserved in heart progenitor cells in Drosophila, Xenopus, zebrafish, chick, and mouse (Harvey 1996). Simple sets of rules have also been proposed that define how a cell is structured (Rafelski and Marshall 2008). However, whether mechanical processes sculpting tissues are conserved across model systems is an open question. At the cellular scale, the most common force generator across metazoans is actomyosin, which generates tensile force sufficient to induce cell shape changes observed across animal species; from apical constriction and anisotropic growth to protrusion and cytokinesis (Lecuit and Lenne 2007; Murrell et al. 2015; Priya and Yap 2015). Furthermore, simple local interactions governed by differential tension or adhesion are capable of triggering cell fate specification and spatial patterning in a variety of developmental contexts (Maitre et al. 2016; Miroshnikova et al. 2018; Wickstrom and Niessen 2018; Priya et al. 2020; Tsai et al. 2020). Indeed, as discussed in this review, morphogenetic systems appear to converge on similar physical principles to yield specific structural motifs. Differential growth triggers buckling deformations in the intestine, heart and brain (Fig. 1A), while stochastic bud branching and termination form elaborate branching architectures in the mammary gland, kidney and prostate (Fig. 4A). Of note, work in non-model systems at key phylogenetic nodes has also identified highly conserved repertoires of cell behaviours, with variation in tissue form arising more from changes in the timing and magnitude of processes than their inherent quality (Steventon et al. 2016; Attardi et al. 2018; Andrews et al. 2021). Morphogenetic systems may thus exploit a shared array of design rules, albeit in different combinations, extents and length scales, to produce the tremendous variety of morphologies observed in a developing embryo (for an evolutionary perspective see Jacob 1977). As a result, while we may focus on a particular process in a particular model system, there is cause to seek general rules of morphogenesis, requiring us to integrate our findings into a broad framework applicable to various species and developmental contexts. We expect that while some mechanical principles will be context-specific, others will transcend length scales and model systems.

## Conclusion

To gain a holistic understanding of how organ form and function emerge, we need to focus on mechanics of morphogenesis *in toto* and across length scales. While one can contend that embryonic morphogenesis is too complex to decode, we propose that with the advent of state-of-art optical, biophysical, and genetic tools we should strive to embrace this complexity. This grand challenge will require a truly interdisciplinary effort combining skills from different areas of biology, physics, and mathematics to reveal the fundamental essence of morphogenesis.

## References

[R1] (2023). What’s next for bioimage analysis?. Nat Methods.

[R2] Agarwal P, Shemesh T, Zaidel-Bar R (2022). Directed cell invasion and asymmetric adhesion drive tissue elongation and turning in C. elegans gonad morphogenesis. Dev Cell.

[R3] Akhtar N, Streuli CH (2013). An integrin-ILK-microtubule network orients cell polarity and lumen formation in glandular epithelium. Nat Cell Biol.

[R4] Alt S, Ganguly P, Salbreux G (2017). Vertex models: From cell mechanics to tissue morphogenesis. Philos Trans R Soc Lond B Biol Sci.

[R5] Alvers AL, Ryan S, Scherz PJ, Huisken J, Bagnat M (2014). Single continuous lumen formation in the zebrafish gut is mediated by smoothened-dependent tissue remodeling. Development.

[R6] Andrews TGR, Pönisch W, Paluch EK, Steventon BJ, Benito-Gutierrez E (2021). Single-cell morphometrics reveals ancestral principles of notochord development. Development.

[R7] Attardi A, Fulton T, Florescu M, Shah G, Muresan L, Lenz MO, Lancaster C, Huisken J, van Oudenaarden A, Steventon B (2018). Neuromesodermal progenitors are a conserved source of spinal cord with divergent growth dynamics. Development.

[R8] Auman HJ, Coleman H, Riley HE, Olale F, Tsai HJ, Yelon D (2007). Functional modulation of cardiac form through regionally confined cell shape changes. PLoS Biol.

[R9] Baer KEv (1828). Über Entwickelungsgeschichte der Thiere: Beobachtung und Reflexion.

[R10] Bagnat M, Cheung ID, Mostov KE, Stainier DY (2007). Genetic control of single lumen formation in the zebrafish gut. Nat Cell Biol.

[R11] Bagnat M, Navis A, Herbstreith S, Brand-Arzamendi K, Curado S, Gabriel S, Mostov K, Huisken J, Stainier DY (2010). Cse1l is a negative regulator of CFTR-dependent fluid secretion. Curr Biol.

[R12] Barbacena P, Dominguez-Cejudo M, Fonseca CG, Gomez-Gonzalez M, Faure LM, Zarkada G, Pena A, Pezzarossa A, Ramalho D, Giarratano Y (2022). Competition for endothelial cell polarity drives vascular morphogenesis in the mouse retina. Dev Cell.

[R13] Barone V, Lang M, Krens SFG, Pradhan SJ, Shamipour S, Sako K, Sikora M, Guet CC, Heisenberg CP (2017). An effective feedback loop between cell-cell contact duration and morphogen signaling determines cell fate. Dev Cell.

[R14] Bassel GW, Smith RS (2016). Quantifying morphogenesis in plants in 4D. Curr Op Plant Biol.

[R15] Bayraktar M, Männer J (2014). Cardiac looping may be driven by compressive loads resulting from unequal growth of the heart and pericardial cavity. Observations on a physical simulation model. Front Physiol.

[R16] Beis D, Bartman T, Jin SW, Scott IC, D’Amico LA, Ober EA, Verkade H, Frantsve J, Field HA, Wehman A (2005). Genetic and cellular analyses of zebrafish atrioventricular cushion and valve development. Development.

[R17] Boezio GLM, Bensimon-Brito A, Piesker J, Guenther S, Helker CSM, Stainier DYR (2020). Endothelial TGF-β signaling instructs smooth muscle cell development in the cardiac outflow tract. eLife.

[R18] Bothma JP, Norstad MR, Alamos S, Garcia HG (2018). LlamaTags: A versatile tool to image transcription factor dynamics in live embryos. Cell.

[R19] Bray SJ (2016). Notch signalling in context. Nat Rev Mol Cell Biol.

[R20] Briggs JA, Weinreb C, Wagner DE, Megason S, Peshkin L, Kirschner MW, Klein AM (2018). The dynamics of gene expression in vertebrate embryogenesis at single-cell resolution. Science.

[R21] Brownfield DG, de Arce AD, Ghelfi E, Gillich A, Desai TJ, Krasnow MA (2022). Alveolar cell fate selection and lifelong maintenance of AT2 cells by FGF signaling. Nat Commun.

[R22] Buckley CE, Ren X, Ward LC, Girdler GC, Araya C, Green MJ, Clark BS, Link BA, Clarke JD (2013). Mirror-symmetric microtubule assembly and cell interactions drive lumen formation in the zebrafish neural rod. EMBO J.

[R23] Butler MB, Short NE, Maniou E, Alexandre P, Greene NDE, Copp AJ, Galea GL (2019). Rho kinase-dependent apical constriction counteracts M-phase apical expansion to enable mouse neural tube closure. J Cell Sci.

[R24] Campas O (2016). A toolbox to explore the mechanics of living embryonic tissues. Semin Cell Dev Biol.

[R25] Caussinus E, Colombelli J, Affolter M (2008). Tip-cell migration controls stalk-cell intercalation during Drosophila tracheal tube elongation. Curr Biol.

[R26] Chan CJ, Heisenberg CP, Hiiragi T (2017). Coordination of morphogenesis and cell-fate specification in development. Curr Biol.

[R27] Chen T, Callan-Jones A, Fedorov E, Ravasio A, Brugues A, Ong HT, Toyama Y, Low BC, Trepat X, Shemesh T (2019). Large-scale curvature sensing by directional actin flow drives cellular migration mode switching. Nat Phys.

[R28] Chow RW, Fukui H, Chan WX, Tan KSJ, Roth S, Duchemin AL, Messaddeq N, Nakajima H, Liu F, Faggianelli-Conrozier N (2022). Cardiac forces regulate zebrafish heart valve delamination by modulating Nfat signaling. PLoS Biol.

[R29] Christodoulou N, Skourides PA (2022). Somitic mesoderm morphogenesis is necessary for neural tube closure during Xenopus development. Front Cell Dev Biol.

[R30] Clark E (2017). Dynamic patterning by the Drosophila pair-rule network reconciles long-germ and short-germ segmentation. PLoS Biol.

[R31] Collinet C, Lecuit T (2021). Programmed and self-organized flow of information during morphogenesis. Nat Rev Mol Cell Biol.

[R32] Conrad L, Runser SVM, Fernando Gomez H, Lang CM, Dumond MS, Sapala A, Schaumann L, Michos O, Vetter R, Iber D (2021). The biomechanical basis of biased epithelial tube elongation in lung and kidney development. Development.

[R33] D'Angelo A, Dierkes K, Carolis C, Salbreux G, Solon J (2019). In vivo force application reveals a fast tissue softening and external friction increase during early embryogenesis. Curr Biol.

[R34] Dahl-Jensen SB, Yennek S, Flasse L, Larsen HL, Sever D, Karremore G, Novak I, Sneppen K, Grapin-Botton A (2018). Deconstructing the principles of ductal network formation in the pancreas. PLoS Biol.

[R35] Davidson L, von Dassow M, Zhou J (2009). Multi-scale mechanics from molecules to morphogenesis. Int J Biochem Cell Biol.

[R36] Davidson LA, Labouesse M (2011). Embryo mechanics: Balancing force production with elastic resistance during morphogenesis. Current Topics in Developmental Biology.

[R37] Davies J (2017). Using synthetic biology to explore principles of development. Development.

[R38] Del Monte-Nieto G, Ramialison M, Adam AAS, Wu B, Aharonov A, D’Uva G, Bourke LM, Pitulescu ME, Chen H, de la Pompa JL (2018). Control of cardiac jelly dynamics by NOTCH1 and NRG1 defines the building plan for trabeculation. Nature.

[R39] Desgrange A, Le Garrec JF, Meilhac SM (2018). Left-right asymmetry in heart development and disease: Forming the right loop. Development.

[R40] Desgrange A, Le Garrec JF, Bernheim S, Bønnelykke TH, Meilhac SM (2020). Transient nodal signaling in left precursors coordinates opposed asymmetries shaping the heart loop. Dev Cell.

[R41] Dong B, Hannezo E, Hayashi S (2014). Balance between apical membrane growth and luminal matrix resistance determines epithelial tubule shape. Cell Rep.

[R42] Duchemin AL, Vignes H, Vermot J (2019). Mechanically activated piezo channels modulate outflow tract valve development through the Yap1 and Klf2-Notch signaling axis. eLife.

[R43] Dullweber T, Erzberger A (2023). Mechanochemical feedback loops in contact-dependent fate patterning. Curr Opin Syst Biol.

[R44] Dunwoodie SL, Wallingford JB (2020). Diseases of development: Leveraging developmental biology to understand human disease. Development.

[R45] Fletcher AG, Osborne JM (2022). Seven challenges in the multiscale modeling of multicellular tissues. WIREs Mech Dis.

[R46] Fletcher DA (2016). Bottom-Up biology: Harnessing engineering to understand nature. Dev Cell.

[R47] Franco CA, Jones ML, Bernabeu MO, Geudens I, Mathivet T, Rosa A, Lopes FM, Lima AP, Ragab A, Collins RT (2015). Dynamic endothelial cell rearrangements drive developmental vessel regression. PLoS Biol.

[R48] Fukui H, Chow RW, Xie J, Foo YY, Yap CH, Minc N, Mochizuki N, Vermot J (2021). Bioelectric signaling and the control of cardiac cell identity in response to mechanical forces. Science.

[R49] Fulton T, Spiess K, Thomson L, Wang Y, Clark B, Hwang S, Paige B, Verd B, Steventon B (2022). Cell rearrangement generates pattern emergence as a function of temporal morphogen exposure. bioRxiv.

[R50] Galea GL, Maniou E, Edwards TJ, Marshall AR, Ampartzidis I, Greene NDE, Copp AJ (2021). Cell non-autonomy amplifies disruption of neurulation by mosaic Vangl2 deletion in mice. Nat Commun.

[R51] Galea GL, Cho YJ, Galea G, Mole MA, Rolo A, Savery D, Moulding D, Culshaw LH, Nikolopoulou E, Greene NDE (2017). Biomechanical coupling facilitates spinal neural tube closure in mouse embryos. Proc Natl Acad Sci.

[R52] Gehrels EW, Chakrabortty B, Perrin ME, Merkel M, Lecuit T (2023). Curvature gradient drives polarized tissue flow in the Drosophila embryo. Proc Natl Acad Sci.

[R53] Gillich A, Julien KR, Brownfield DG, Travaglini KJ, Metzger RJ, Krasnow MA (2021). Alveoli form directly by budding led by a single epithelial cell. biorxiv.

[R54] Gilmour D, Rembold M, Leptin M (2017). From morphogen to morphogenesis and back. Nature.

[R55] Gómez-González M, Latorre E, Arroyo M, Trepat X (2020). Measuring mechanical stress in living tissues. Nat Revs Phys.

[R56] Good M, Trepat X (2018). Cell parts to complex processes, from the bottom up. Nature.

[R57] Goodwin K, Nelson CM (2020). Branching morphogenesis. Development.

[R58] Goodwin K, Nelson CM (2021). Mechanics of development. Dev Cell.

[R59] Goodwin K, Mao S, Guyomar T, Miller E, Radisky DC, Kosmrlj A, Nelson CM (2019). Smooth muscle differentiation shapes domain branches during mouse lung development. Development.

[R60] Gosak M, Milojevic M, Duh M, Skok K, Perc M (2022). Networks behind the morphology and structural design of living systems. Phys Life Rev.

[R61] Grego-Bessa J, Gómez-Apiñaniz P, Prados B, Gómez MJ, MacGrogan D, Pompa JLdl (2023). Neuregulin-1 regulates cardiomyocyte dynamics, cell cycle progression, and maturation during ventricular chamber morphogenesis. bioRxiv.

[R62] Gunawan F, Priya R, Stainier DYR (2021). Sculpting the heart: Cellular mechanisms shaping valves and trabeculae. Curr Opin Cell Biol.

[R63] Gunawan F, Gentile A, Fukuda R, Tsedeke AT, Jiménez-Amilburu V, Ramadass R, Iida A, Sehara-Fujisawa A, Stainier DY (2019). Focal adhesions are essential to drive zebrafish heart valve morphogenesis. J Cell Biol.

[R64] Gupta V, Poss KD (2012). Clonally dominant cardiomyocytes direct heart morphogenesis. Nature.

[R65] Haack T, Schneider M, Schwendele B, Renault AD (2014). Drosophila heart cell movement to the midline occurs through both cell autonomous migration and dorsal closure. Dev Biol.

[R66] Haddon C, Lewis J (1996). Early ear development in the embryo of the zebrafish, Danio rerio. J Comp Neurol.

[R67] Hadjivasiliou Z, Hunter G, Kornberg T (2022). Talking to your neighbors across scales: Long-distance Notch signaling during patterning. Current Topics in Developmental Biology.

[R68] Haeckel E (1866). Generelle morphologie der organismen Allgemeine grundzüge der organischen formen-wissenschaft, mechanisch begründet durch die von Charles Darwin reformirte descendenztheorie.

[R69] Haigo SL, Hildebrand JD, Harland RM, Wallingford JB (2003). Shroom induces apical constriction and is required for hingepoint formation during neural tube closure. Curr Biol.

[R70] Hamant O, Saunders TE (2020). Shaping organs: Shared structural principles across kingdoms. Annu Rev Cell Dev Biol.

[R71] Hannezo E, Heisenberg CP (2019). Mechanochemical feedback loops in development and disease. Cell.

[R72] Hannezo E, Dong B, Recho P, Joanny JF, Hayashi S (2015). Cortical instability drives periodic supracellular actin pattern formation in epithelial tubes. Proc Natl Acad Sci.

[R73] Hannezo E, Scheele CLGJ, Moad M, Drogo N, Heer R, Sampogna RV, van Rheenen J, Simons BD (2017). A unifying theory of branching morphogenesis. Cell.

[R74] Harunaga J, Hsu JC, Yamada KM (2011). Dynamics of salivary gland morphogenesis. J Dent Res.

[R75] Harunaga JS, Doyle AD, Yamada KM (2014). Local and global dynamics of the basement membrane during branching morphogenesis require protease activity and actomyosin contractility. Dev Biol.

[R76] Harvey RP (1996). NK-2 homeobox genes and heart development. Dev Biol.

[R77] Hashimoto H, Munro E (2019). Differential expression of a classic cadherin directs tissue-level contractile asymmetry during neural tube closure. Dev Cell.

[R78] Hashimoto H, Robin FB, Sherrard KM, Munro EM (2015). Sequential contraction and exchange of apical junctions drives zippering and neural tube closure in a simple chordate. Dev Cell.

[R79] Heermann S, Schütz L, Lemke S, Krieglstein K, Wittbrodt J (2015). Eye morphogenesis driven by epithelial flow into the optic cup facilitated by modulation of bone morphogenetic protein. eLife.

[R80] Helker CS, Schuermann A, Karpanen T, Zeuschner D, Belting HG, Affolter M, Schulte-Merker S, Herzog W (2013). The zebrafish common cardinal veins develop by a novel mechanism: Lumen ensheathment. Development.

[R81] Hirashima T, Matsuda M (2024). ERK-mediated curvature feedback regulates branching morphogenesis in lung epithelial tissue. Curr Biol.

[R82] Hofer M, Lutolf MP (2021). Engineering organoids. Nat Rev Mater.

[R83] Hoijman E, Rubbini D, Colombelli J, Alsina B (2015). Mitotic cell rounding and epithelial thinning regulate lumen growth and shape. Nat Commun.

[R84] Houtekamer RM, van der Net MC, Maurice MM, Gloerich M (2022). Mechanical forces directing intestinal form and function. Curr Biol.

[R85] Hoyle C, Brown NA, Wolpert L (1992). Development of left/right handedness in the chick heart. Development.

[R86] Huang CK, Yong X, She DT, Lim CT (2023). Surface curvature and basal hydraulic stress induce spatial bias in cell extrusion. eLife.

[R87] Ingber DE (2005). Mechanical control of tissue growth: Function follows form. Proc Natl Acad Sci.

[R88] Ishihara K, Mukherjee A, Gromberg E, Brugués J, Tanaka EM, Jülicher F (2022). Topological morphogenesis of neuroepithelial organoids. Nat Phys.

[R89] Itoh K, Ossipova O, Sokol SY (2014). GEF-H1 functions in apical constriction and cell intercalations and is essential for vertebrate neural tube closure. J Cell Sci.

[R90] Jacob F (1977). Evolution and tinkering. Science.

[R91] Jaeger J, Surkova S, Blagov M, Janssens H, Kosman D, Kozlov KN, Myasnikova E, Vanario-Alonso CE, Samsonova M (2004). Dynamic control of positional information in the early Drosophila embryo. Nature.

[R92] Jimenez-Amilburu V, Rasouli SJ, Staudt DW, Nakajima H, Chiba A, Mochizuki N, Stainier DYR (2016). In vivo visualization of cardiomyocyte apicobasal polarity reveals epithelial to mesenchymal-like transition during cardiac trabeculation. Cell Rep.

[R93] Karzbrun E, Kshirsagar A, Cohen SR, Hanna JH, Reiner O (2018). Human brain organoids on a chip reveal the physics of folding. Nat Phys.

[R94] Karzbrun E, Khankhel AH, Megale HC, Glasauer SMK, Wyle Y, Britton G, Warmflash A, Kosik KS, Siggia ED, Shraiman BI (2021). Human neural tube morphogenesis in vitro by geometric constraints. Nature.

[R95] Kaster T, Sack I, Samani A (2011). Measurement of the hyperelastic properties of ex vivo brain tissue slices. J Biomech.

[R96] Kawahira N, Ohtsuka D, Kida N, Hironaka K-i, Morishita Y (2020). Quantitative analysis of 3D tissue deformation reveals key cellular mechanism associated with initial heart looping. Cell Rep.

[R97] Keller R (2012). Developmental biology. Physical biology returns to morphogenesis. Science.

[R98] Khoromskaia D, Salbreux G (2023). Active morphogenesis of patterned epithelial shells. Elife.

[R99] Kicheva A, Briscoe J (2023). Control of tissue development by morphogens. Annu Rev Cell Dev Biol.

[R100] Kim HY, Pang MF, Varner VD, Kojima L, Miller E, Radisky DC, Nelson CM (2015). Localized smooth muscle differentiation is essential for epithelial bifurcation during branching morphogenesis of the mammalian lung. Dev Cell.

[R101] King TR, Kramer J, Cheng YS, Swope D, Kramer SG (2021). Enabled/VASP is required to mediate proper sealing of opposing cardioblasts during Drosophila dorsal vessel formation. Dev Dyn.

[R102] Lancino M, Majello S, Herbert S, De Chaumont F, Tinevez JY, Olivo-Marin JC, Herbomel P, Schmidt A (2018). Anisotropic organization of circumferential actomyosin characterizes hematopoietic stem cells emergence in the zebrafish. eLife.

[R103] Latorre E, Kale S, Casares L, Gomez-Gonzalez M, Uroz M, Valon L, Nair RV, Garreta E, Montserrat N, Del Campo A (2018). Active superelasticity in three-dimensional epithelia of controlled shape. Nature.

[R104] Le Garrec JF, Domínguez JN, Desgrange A, Ivanovitch KD, Raphaël E, Bangham JA, Torres M, Coen E, Mohun TJ, Meilhac SM (2017). A predictive model of asymmetric morphogenesis from 3D reconstructions of mouse heart looping dynamics. eLife.

[R105] Lecuit T, Lenne PF (2007). Cell surface mechanics and the control of cell shape, tissue patterns and morphogenesis. Nat Rev Mol Cell Biol.

[R106] Lenne PF, Munro E, Heemskerk I, Warmflash A, Bocanegra-Moreno L, Kishi K, Kicheva A, Long Y, Fruleux A, Boudaoud A (2021). Roadmap for the multiscale coupling of biochemical and mechanical signals during development. Phys Biol.

[R107] Li B, Brusman L, Dahlka J, Niswander LA (2022). TMEM132A ensures mouse caudal neural tube closure and regulates integrin-based mesodermal migration. Development.

[R108] Li J, Economou AD, Vacca B, Green JBA (2020). Epithelial invagination by a vertical telescoping cell movement in mammalian salivary glands and teeth. Nat Commun.

[R109] Li J, Wang Z, Chu Q, Jiang K, Li J, Tang N (2018). The strength of mechanical forces determines the differentiation of alveolar epithelial cells. Dev Cell.

[R110] Liu J, Bressan M, Hassel D, Huisken J, Staudt D, Kikuchi K, Poss KD, Mikawa T, Stainier DY (2010). A dual role for ErbB2 signaling in cardiac trabeculation. Development.

[R111] Llinares-Benadero C, Borrell V (2019). Deconstructing cortical folding: Genetic, cellular and mechanical determinants. Nat Rev Neurosci.

[R112] Luciano M, Xue SL, De Vos WH, Redondo-Morata L, Surin M, Lafont F, Hannezo E, Gabriele S (2021). Cell monolayers sense curvature by exploiting active mechanics and nuclear mechanoadaptation. Nat Phys.

[R113] Maitre JL, Turlier H, Illukkumbura R, Eismann B, Niwayama R, Nedelec F, Hiiragi T (2016). Asymmetric division of contractile domains couples cell positioning and fate specification. Nature.

[R114] Maroudas-Sacks Y, Keren K (2021). Mechanical patterning in animal morphogenesis. Annu Revf Cell Dev Biol.

[R115] Martyn I, Gartner ZJ (2021). Expanding the boundaries of synthetic development. Dev Biol.

[R116] McDole K, Guignard L, Amat F, Berger A, Malandain G, Royer LA, Turaga SC, Branson K, Keller PJ (2018). In toto imaging and reconstruction of post-implantation mouse development at the single-cell level. Cell.

[R117] Medioni C, Astier M, Zmojdzian M, Jagla K, Semeriva M (2008). Genetic control of cell morphogenesis during Drosophila melanogaster cardiac tube formation. J Cell Biol.

[R118] Melnick M, Jaskoll T (2000). Mouse submandibular gland morphogenesis: a paradigm for embryonic signal processing. Crit Rev Oral Biol Med.

[R119] Menshykau D, Michos O, Lang C, Conrad L, McMahon AP, Iber D (2019). Image-based modeling of kidney branching morphogenesis reveals GDNF-RET based Turing-type mechanism and pattern-modulating WNT11 feedback. Nat Commun.

[R120] Messal HA, Alt S, Ferreira RMM, Gribben C, Wang VM, Cotoi CG, Salbreux G, Behrens A (2019). Tissue curvature and apicobasal mechanical tension imbalance instruct cancer morphogenesis. Nature.

[R121] Metzger RJ, Klein OD, Martin GR, Krasnow MA (2008). The branching programme of mouse lung development. Nature.

[R122] Metzger RJ, Krasnow MA (1999). Genetic control of branching morphogenesis. Science.

[R123] Mickoleit M, Schmid B, Weber M, Fahrbach FO, Hombach S, Reischauer S, Huisken J (2014). High-resolution reconstruction of the beating zebrafish heart. Nat Methods.

[R124] Miroshnikova YA, Le HQ, Schneider D, Thalheim T, Rubsam M, Bremicker N, Polleux J, Kamprad N, Tarantola M, Wang I (2018). Adhesion forces and cortical tension couple cell proliferation and differentiation to drive epidermal stratification. Nat Cell Biol.

[R125] Mitchell NP, Cislo DJ, Shankar S, Lin Y, Shraiman BI, Streichan SJ (2022). Visceral organ morphogenesis via calcium-patterned muscle constrictions. eLife.

[R126] Moreno-Mármol T, Ledesma-Terrón M, Tabanera N, Martin-Bermejo MJ, Cardozo MJ, Cavodeassi F, Bovolenta P (2021). Stretching of the retinal pigment epithelium contributes to zebrafish optic cup morphogenesis. eLife.

[R127] Morishita Y, Hironaka KI, Lee SW, Jin T, Ohtsuka D (2017). Reconstructing 3D deformation dynamics for curved epithelial sheet morphogenesis from positional data of sparsely-labeled cells. Nat Commun.

[R128] Mosaliganti KR, Swinburne IA, Chan CU, Obholzer ND, Green AA, Tanksale S, Mahadevan L, Megason SG (2019). Size control of the inner ear via hydraulic feedback. Elife.

[R129] Munjal A, Hannezo E, Tsai TY, Mitchison TJ, Megason SG (2021). Extracellular hyaluronate pressure shaped by cellular tethers drives tissue morphogenesis. Cell.

[R130] Murrell M, Oakes PW, Lenz M, Gardel ML (2015). Forcing cells into shape: The mechanics of actomyosin contractility. Nat Rev Mol Cell Biol.

[R131] Nelson CM (2016). On buckling morphogenesis. J Biomech Eng.

[R132] Nelson CM, Gleghorn JP (2012). Sculpting organs: Mechanical regulation of tissue development. Annu Rev Biomed Eng.

[R133] Nelson CM, Vanduijn MM, Inman JL, Fletcher DA, Bissell MJ (2006). Tissue geometry determines sites of mammary branching morphogenesis in organotypic cultures. Science.

[R134] Nematbakhsh A, Levis M, Kumar N, Chen W, Zartman JJ, Alber M (2020). Epithelial organ shape is generated by patterned actomyosin contractility and maintained by the extracellular matrix. PLoS Comput Biol.

[R135] Nerger BA, Jaslove JM, Elashal HE, Mao S, Kosmrlj A, Link AJ, Nelson CM (2021). Local accumulation of extracellular matrix regulates global morphogenetic patterning in the developing mammary gland. Curr Biol.

[R136] Nerurkar NL, Mahadevan L, Tabin CJ (2017). BMP signaling controls buckling forces to modulate looping morphogenesis of the gut. Proc Natl Acad Sci.

[R137] Nicolás-Pérez M, Kuchling F, Letelier J, Polvillo R, Wittbrodt J, Martínez-Morales JR (2016). Analysis of cellular behavior and cytoskeletal dynamics reveal a constriction mechanism driving optic cup morphogenesis. eLife.

[R138] Nikolopoulou E, Galea GL, Rolo A, Greene ND, Copp AJ (2017). Neural tube closure: cellular, molecular and biomechanical mechanisms. Development.

[R139] Nikolopoulou E, Hirst CS, Galea G, Venturini C, Moulding D, Marshall AR, Rolo A, De Castro SCP, Copp AJ, Greene NDE (2019). Spinal neural tube closure depends on regulation of surface ectoderm identity and biomechanics by Grhl2. Nat Commun.

[R140] Noël ES, Verhoeven M, Lagendijk AK, Tessadori F, Smith K, Choorapoikayil S, den Hertog J, Bakkers J (2013). A Nodal-independent and tissue-intrinsic mechanism controls heart-looping chirality. Nature Commun.

[R141] Nogawa H, Morita K, Cardoso WV (1998). Bud formation precedes the appearance of differential cell proliferation during branching morphogenesis of mouse lung epithelium in vitro. Dev Dyn.

[R142] Norden C (2023). A fish eye view: Retinal morphogenesis from optic cup to neuronal lamination. Annu Rev Cell Dev Biol.

[R143] Nowotschin S, Hadjantonakis AK, Campbell K (2019). The endoderm: A divergent cell lineage with many commonalities. Development.

[R144] Ochoa-Espinosa A, Harmansa S, Caussinus E, Affolter M (2017). Myosin II is not required for Drosophila tracheal branch elongation and cell intercalation. Development.

[R145] Odell GM, Oster G, Alberch P, Burnside B (1981). The mechanical basis of morphogenesis: I. Epithelial folding and invagination. Dev Biol.

[R146] Okuda S, Miura T, Inoue Y, Adachi T, Eiraku M (2018). Combining Turing and 3D vertex models reproduces autonomous multicellular morphogenesis with undulation, tubulation, and branching. Sci Rep.

[R147] Oster GF, Murray JD, Harris AK (1983). Mechanical aspects of mesenchymal morphogenesis. Development.

[R148] Ozturk-Colak A, Moussian B, Araujo SJ (2016). Drosophila chitinous aECM and its cellular interactions during tracheal development. Dev Dyn.

[R149] Palavalli A, Tizón-Escamilla N, Rupprecht JF, Lecuit T (2021). Deterministic and stochastic rules of branching govern dendrite morphogenesis of sensory neurons. Curr Biol.

[R150] Palmer M, Nerger BA, Goodwin K, Sudhakar A, Lemke SB, Ravindran PT, Toettcher J, Košmrlj A, Nelson CM (2021). Stress ball morphogenesis: How the lizard builds its lung. Sci Adv.

[R151] Peck AL (1953). Generation of Animals.

[R152] Peshkovsky C, Totong R, Yelon D (2011). Dependence of cardiac trabeculation on neuregulin signaling and blood flow in zebrafish. Dev Dyn.

[R153] Petridou NI, Heisenberg CP (2019). Tissue rheology in embryonic organization. EMBO J.

[R154] Pijuan-Sala B, Griffiths JA, Guibentif C, Hiscock TW, Jawaid W, Calero-Nieto FJ, Mulas C, Ibarra-Soria X, Tyser RCV, Ho DLL (2019). A single-cell molecular map of mouse gastrulation and early organogenesis. Nature.

[R155] Prahl LS, Viola JM, Liu J, Hughes AJ (2023). The developing murine kidney actively negotiates geometric packing conflicts to avoid defects. Dev Cell.

[R156] Prevedel R, Diz-Muñoz A, Ruocco G, Antonacci G (2019). Brillouin microscopy: An emerging tool for mechanobiology. Nat Methods.

[R157] Priya R, Allanki S, Gentile A, Mansingh S, Uribe V, Maischein HM, Stainier DYR (2020). Tension heterogeneity directs form and fate to pattern the myocardial wall. Nature.

[R158] Priya R, Yap AS (2015). Active tension: The role of cadherin adhesion and signaling in generating junctional contractility. Curr Top Dev Biol.

[R159] Qi J, Rittershaus A, Priya R, Mansingh S, Stainier DYR, Helker CSM (2022). Apelin signaling dependent endocardial protrusions promote cardiac trabeculation in zebrafish. eLife.

[R160] Rafelski SM, Marshall WF (2008). Building the cell: Design principles of cellular architecture. Nat Rev Mol Cell Biol.

[R161] Rasmussen JP, Reddy SS, Priess JR (2012). Laminin is required to orient epithelial polarity in the C. elegans pharynx. Development.

[R162] Rasouli SJ, Stainier DYR (2017). Regulation of cardiomyocyte behavior in zebrafish trabeculation by Neuregulin 2a signaling. Nat Commun.

[R163] Regot S, Hughey JJ, Bajar BT, Carrasco S, Covert MW (2014). High-sensitivity measurements of multiple kinase activities in live single cells. Cell.

[R164] Richman DP, Stewart RM, Hutchinson J, Caviness VS (1975). Mechanical model of brain convolutional development. Science.

[R165] Rossi G, Manfrin A, Lutolf MP (2018). Progress and potential in organoid research. Nat Rev Genet.

[R166] Samakovlis C, Hacohen N, Manning G, Sutherland DC, Guillemin K, Krasnow MA (1996). Development of the Drosophila tracheal system occurs by a series of morphologically distinct but genetically coupled branching events. Development.

[R167] Samsa LA, Yang B, Liu J (2013). Embryonic cardiac chamber maturation: Trabeculation, conduction, and cardiomyocyte proliferation. Am J Med Genet C Semin Med Genet.

[R168] Santiago-Martinez E, Soplop NH, Patel R, Kramer SG (2008). Repulsion by Slit and Roundabout prevents Shotgun/E-cadherin-mediated cell adhesion during Drosophila heart tube lumen formation. J Cell Biol.

[R169] Saunders TE, Ingham PW (2019). Open questions: How to get developmental biology into shape?. BMC Biol.

[R170] Savin T, Kurpios NA, Shyer AE, Florescu P, Liang H, Mahadevan L, Tabin CJ (2011). On the growth and form of the gut. Nature.

[R171] Saw TB, Doostmohammadi A, Nier V, Kocgozlu L, Thampi S, Toyama Y, Marcq P, Lim CT, Yeomans JM, Ladoux B (2017). Topological defects in epithelia govern cell death and extrusion. Nature.

[R172] Scheele CL, Hannezo E, Muraro MJ, Zomer A, Langedijk NS, van Oudenaarden A, Simons BD, van Rheenen J (2017). Identity and dynamics of mammary stem cells during branching morphogenesis. Nature.

[R173] Shaya O, Binshtok U, Hersch M, Rivkin D, Weinreb S, Amir-Zilberstein L, Khamaisi B, Oppenheim O, Desai RA, Goodyear RJ (2017). Cell-Cell contact area affects notch signaling and notch-dependent patterning. Dev Cell.

[R174] Shi Y, Yao J, Xu G, Taber LA (2014). Bending of the looping heart: Differential growth revisited. J Biomech Eng.

[R175] Shiraishi K, Shah PP, Morley MP, Loebel C, Santini GT, Katzen J, Basil MC, Lin SM, Planer JD, Cantu E (2023). Biophysical forces mediated by respiration maintain lung alveolar epithelial cell fate. Cell.

[R176] Shu T, Szórádi T, Kidiyoor GR, Xie Y, Herzog NL, Bazley A, Bonucci M, Keegan S, Saxena S, Ettefa F (2022). nucGEMs probe the biophysical properties of the nucleoplasm. bioRxiv.

[R177] Shyer AE, Tallinen T, Nerurkar NL, Wei Z, Gil ES, Kaplan DL, Tabin CJ, Mahadevan L (2013). Villification: How the gut gets its villi. Science.

[R178] Sidhaye J, Norden C (2017). Concerted action of neuroepithelial basal shrinkage and active epithelial migration ensures efficient optic cup morphogenesis. eLife.

[R179] Sigurbjornsdottir S, Mathew R, Leptin M (2014). Molecular mechanisms of de novo lumen formation. Nat Rev Mol Cell Biol.

[R180] Silver BB, Wolf AE, Lee J, Pang MF, Nelson CM (2020). Epithelial tissue geometry directs emergence of bioelectric field and pattern of proliferation. Mol Biol Cell.

[R181] Smith JL, Schoenwolf GC (1988). Role of cell-cycle in regulating neuroepithelial cell shape during bending of the chick neural plate. Cell Tissue Res.

[R182] Staudt DW, Liu J, Thorn KS, Stuurman N, Liebling M, Stainier DY (2014). High-resolution imaging of cardiomyocyte behavior reveals two distinct steps in ventricular trabeculation. Development.

[R183] Steventon B, Duarte F, Lagadec R, Mazan S, Nicolas JF, Hirsinger E (2016). Species-specific contribution of volumetric growth and tissue convergence to posterior body elongation in vertebrates. Development.

[R184] Stooke-Vaughan GA, Campas O (2018). Physical control of tissue morphogenesis across scales. Curr Opin Genet Dev.

[R185] Stower M, Zhou F, Hathrell H, Yeung J, Thowfeequ S, Godwin J, Schneider F, Lagerholm C, Fritzsche M, Thiyagalingam J (2023). Single-cell phenomics reveals behavioural and mechanical heterogeneities underpinning collective migration during mouse anterior patterning. bioRxiv.

[R186] Striedter GF, Srinivasan S, Monuki ES (2015). Cortical folding: When, where, how, and why?. Annu Rev Neurosci.

[R187] Sugimura K, Lenne PF, Graner F (2016). Measuring forces and stresses in situ in living tissues. Development.

[R188] Taber LA (2006). Biophysical mechanisms of cardiac looping. Int J Dev Biol.

[R189] Tallinen T, Chung JY, Biggins JS, Mahadevan L (2014). Gyrification from constrained cortical expansion. Proc Natl Acad Sci.

[R190] Tallinen T, Chung JY, Rousseau F, Girard N, Lefèvre J, Mahadevan L (2016). On the growth and form of cortical convolutions. Nat Phys.

[R191] Tang Z, Hu Y, Wang Z, Jiang K, Zhan C, Marshall WF, Tang N (2018). Mechanical forces program the orientation of cell division during airway tube morphogenesis. Dev Cell.

[R192] Tessadori F, Tsingos E, Colizzi ES, Kruse F, van den Brink SC, van den Boogaard M, Christoffels VM, Merks RMH, Bakkers J (2021). Twisting of the zebrafish heart tube during cardiac looping is a tbx5-dependent and tissue-intrinsic process. eLife.

[R193] Thompson DAW (1917). On Growth and Form.

[R194] Thomson JA (1917a). On Growth and Form. Nature.

[R195] Tonning A, Hemphala J, Tang E, Nannmark U, Samakovlis C, Uv A (2005). A transient luminal chitinous matrix is required to model epithelial tube diameter in the Drosophila trachea. Dev Cell.

[R196] Torres-Sanchez A, Winter Kerr, Salbreux G (2022). Interacting active surfaces: A model for three-dimensional cell aggregates. PLoS Comput Biol.

[R197] Tozluoǧlu M, Duda M, Kirkland NJ, Barrientos R, Burden JJ, Muñoz JJ, Mao Y (2019). Planar differential growth rates initiate precise fold positions in complex epithelia. Dev Cell.

[R198] Tozluoǧlu M, Mao Y (2020). On folding morphogenesis, a mechanical problem. Phil Trans R Soc B.

[R199] Träber N, Uhlmann K, Girardo S, Kesavan G, Wagner K, Friedrichs J, Goswami R, Bai K, Brand M, Werner C (2019). Polyacrylamide bead sensors for in vivo quantification of cell-scale stress in zebrafish development. Sci Rep.

[R200] Tremblay KD, Kaestner KH (2010). Chapter 1 - Formation of the murine endoderm: Lessons from the mouse, frog, fish, and chick. Progress in Molecular Biology and Translational Science.

[R201] Trepat X, Sahai E (2018). Mesoscale physical principles of collective cell organization. Na Phys.

[R202] Tsai TY, Sikora M, Xia P, Colak-Champollion T, Knaut H, Heisenberg CP, Megason SG (2020). An adhesion code ensures robust pattern formation during tissue morphogenesis. Science.

[R203] Ucar MC, Kamenev D, Sunadome K, Fachet D, Lallemend F, Adameyko I, Hadjab S, Hannezo E (2021). Theory of branching morphogenesis by local interactions and global guidance. Nat Commun.

[R204] Uriu K, Liao BK, Oates AC, Morelli LG (2021). From local resynchronization to global pattern recovery in the zebrafish segmentation clock. eLife.

[R205] Varner VD, Gleghorn JP, Miller E, Radisky DC, Nelson CM (2015). Mechanically patterning the embryonic airway epithelium. Proc Natl Acad Sci.

[R206] Varner VD, Nelson CM (2014). Cellular and physical mechanisms of branching morphogenesis. Development.

[R207] Veenvliet JV, Lenne PF, Turner DA, Nachman I, Trivedi V (2021). Sculpting with stem cells: How models of embryo development take shape. Development.

[R208] Vercurysse E, Brückner DB, Gómez-González M, Luciano M, Kalukula Y, Rossetti L, Trepat X, Hannezo E, Gabriele S (2022). Geometry-driven migration efficiency of minimal cell clusters. bioRxiv.

[R209] Verd B, Clark E, Wotton KR, Janssens H, Jiménez-Guri E, Crombach A, Jaeger J (2018). A damped oscillator imposes temporal order on posterior gap gene expression in Drosophila. PLoS Biol.

[R210] Vermot J, Forouhar AS, Liebling M, Wu D, Plummer D, Gharib M, Fraser SE (2009). Reversing blood flows act through klf2a to ensure normal valvulogenesis in the developing heart. PLoS Biol.

[R211] Vignes H, Vagena-Pantoula C, Vermot J (2022). Mechanical control of tissue shape: Cell-extrinsic and -intrinsic mechanisms join forces to regulate morphogenesis. Semin Cell Dev Biol.

[R212] Villedieu A, Alpar L, Gaugué I, Joudat A, Graner F, Bosveld F, Bellaïche Y (2023). Homeotic compartment curvature and tension control spatiotemporal folding dynamics. Nat Commun.

[R213] Vogt W (1929). Gestaltungsanalyse am Amphibienkeim mit Örtlicher Vitalfärbung : II. Teil. Gastrulation und Mesodermbildung bei Urodelen und Anuren. Wilhelm Roux Arch Entwickl Mech Org.

[R214] Wagner DE, Weinreb C, Collins ZM, Briggs JA, Megason SG, Klein AM (2018). Single-cell mapping of gene expression landscapes and lineage in the zebrafish embryo. Science.

[R215] Wallingford JB (2019). The 200-year effort to see the embryo. Science.

[R216] Walton KD, Freddo AM, Wang S, Gumucio DL (2016a). Generation of intestinal surface: An absorbing tale. Development.

[R217] Walton KD, Whidden M, Kolterud Å, Shoffner KS, Czerwinski MJ, Kushwaha J, Parmar N, Chandhrasekhar D, Freddo AM, Schnell S (2016b). Villification in the mouse: Bmp signals control intestinal villus patterning. Development.

[R218] Wang S, Matsumoto K, Lish SR, Cartagena-Rivera AX, Yamada KM (2021). Budding epithelial morphogenesis driven by cell-matrix versus cell-cell adhesion. Cell.

[R219] Wang X, Merkel M, Sutter LB, Erdemci-Tandogan G, Manning ML, Kasza KE (2020). Anisotropy links cell shapes to tissue flow during convergent extension. Proc Natl Acad Sci.

[R220] Wickstrom SA, Niessen CM (2018). Cell adhesion and mechanics as drivers of tissue organization and differentiation: Local cues for large scale organization. Curr Opin Cell Biol.

[R221] Wilcockson SG, Guglielmi L, Rodriguez PA, Amoyel M, Hill CS (2022). An improved Erk biosensor detects oscillatory Erk dynamics driven by mitotic erasure during early development. Dev Cell.

[R222] Xia P, Gutl D, Zheden V, Heisenberg CP (2019). Lateral inhibition in cell specification mediated by mechanical signals modulating TAZ activity. Cell.

[R223] Xu H, Sun M, Zhao X (2017). Turing mechanism underlying a branching model for lung morphogenesis. PLoS One.

[R224] Ybot-Gonzalez P, Cogram P, Gerrelli D, Copp AJ (2002). Sonic hedgehog and the molecular regulation of mouse neural tube closure. Development.

[R225] Zhang S, Amourda C, Garfield D, Saunders TE (2018). Selective filopodia adhesion ensures robust cell matching in the drosophila heart. Dev Cell.

[R226] Zhang S, Teng X, Toyama Y, Saunders TE (2020). Periodic oscillations of myosin-II mechanically proofread cell-cell connections to ensure robust formation of the cardiac vessel. Curr Biol.

[R227] Zhou CJ, Ji Y, Reynolds K, McMahon M, Garland MA, Zhang S, Sun B, Gu R, Islam M, Liu Y (2020). Non-neural surface ectodermal rosette formation and F-actin dynamics drive mammalian neural tube closure. Biochem Biophys Res Commun.

